# Galantamine improves glycemic control and diabetic nephropathy in Lepr^db/db^ mice

**DOI:** 10.1038/s41598-023-42665-2

**Published:** 2023-09-20

**Authors:** Qinghe Meng, Julia Ma, Liye Suo, Napat Pruekprasert, Prithi Chakrapani, Robert N. Cooney

**Affiliations:** 1 Department of Surgery, State University of New York (SUNY), Upstate Medical University, 750 E Adams St., Suite 8141, Syracuse, NY 13210 USA; 2 Department of Pathology, State University of New York (SUNY), Upstate Medical University, Syracuse, NY USA

**Keywords:** Kidney, Kidney diseases, Endocrine system and metabolic diseases, Diabetes, Obesity, Endocrinology, Nephrology

## Abstract

Galantamine, a centrally acting acetylcholinesterase inhibitor, has been shown to attenuate inflammation and insulin resistance in patients with metabolic syndrome. We investigated the effects of galantamine on glycemic control and development of diabetic nephropathy (DN) in Lepr^db/db^ mice. Galantamine significantly reduced food intake, body weight, blood glucose and HbA1c levels. Insulin resistance (HOMA-IR, QUICKI), HOMA-β and elevations in plasma inflammatory cytokine levels (TNF-α, IL-6 and HMGB-1) were all attenuated by galantamine. Galantamine also ameliorated diabetes-induced kidney injury as evidenced by improvements in renal function (BUN, creatinine, albuminuria), histologic injury and apoptosis. Improved glycemic control and nephropathy were associated with increased circulating GLP-1, decreased renal P-38 MAPK and caspase-1 activation and reduced SGLT-2 expression. These findings provide insights into the mechanisms by which galantamine improves glycemic control and attenuates DN in the Lepr^db/db^ mouse model.

## Introduction

According to the WHO, approximately 422 million people worldwide suffer from diabetes. The majority suffer from type 2 diabetes mellitus (T2DM) which is associated with obesity, insulin resistance, hyperglycemia^1^ and beta cell loss resulting in decreased insulin secretion^2^. Over time many patients with T2DM develop severe complications including blindness due to retinopathy, cardiovascular disease, and diabetic nephropathy (DN) causing end stage renal disease (ESRD) which increases their risk of death^3^. The development of albuminuria is the hallmark of DN^4^, and early treatment focuses on glycemic control and blood pressure management^5^. The pathogenesis of DN is multifactorial with hyperglycemia, inflammation, oxidative stress, hemodynamic disturbances, and apoptotic cell death as contributing factors^6–8^. Diabetic renal injury is characterized histologically by mesangial matrix expansion, increased glomerular size and podocyte loss^9^. The Lepr^db/db^ (db/db) mouse is commonly used for studying T2DM and DN. The db/db mouse is hyperphagic, obese and hyperinsulinemic due to leptin deficiency^10^. Furthermore, changes in the db/db mouse kidney over time closely resemble DN in the human condition^11–13^. Reductions in postprandial circulating GLP-1 levels have been reported in db/db mice^14^. In addition, increased expressions of renal SGLT-2 and impaired function of SGLT-2 in db/db mice have been demonstrated from multiple studies^15–19^.

Recent studies suggest the vagus nerve and cholinergic anti-inflammatory pathway (CAP) are important in attenuating experimental acute kidney injury^20^. The vagus is a “mixed” nerve containing both afferent and efferent fibers which runs from the brainstem through the chest and abdominal cavities innervating multiple viscera. Vagal afferent fibers sense intra-peritoneal inflammation and signal this to the brainstem. Reflex activation of the vagal efferent in the brainstem reduces inflammation by activating the α7nAChR in peripheral tissues. A number of α7nAChR receptor agonists, including GTS-21, an α7 nicotinic acetylcholine receptor (α7nAChR) agonist can improve glycemic control and DN in the db/db mouse^21–23^.

Galantamine acts via central muscarinic receptors to suppress systemic inflammation by activating vagal efferent and peripheral α7nAChRs^24^. The current study examines the effects of galantamine on food intake, body weight, glycemic control, and the development of DN in the db/db mouse model. Our results provide evidence galantamine impacts T2DM and DN through multiple mechanisms in the db/db mouse model including reduced food intake and weight loss, improvements in glycemic control and insulin resistance, decreasing systemic inflammation and renal apoptosis in part by modulating activity of the p38 MAPK and Caspase-1 pathways. More importantly, it provides evidence to support the CAP as a therapeutic target for T2DM and prevention of diabetic complications like DN.

## Results

### Effects of galantamine on food intake, body weight, glycemic control and inflammation

**Figure 1:**
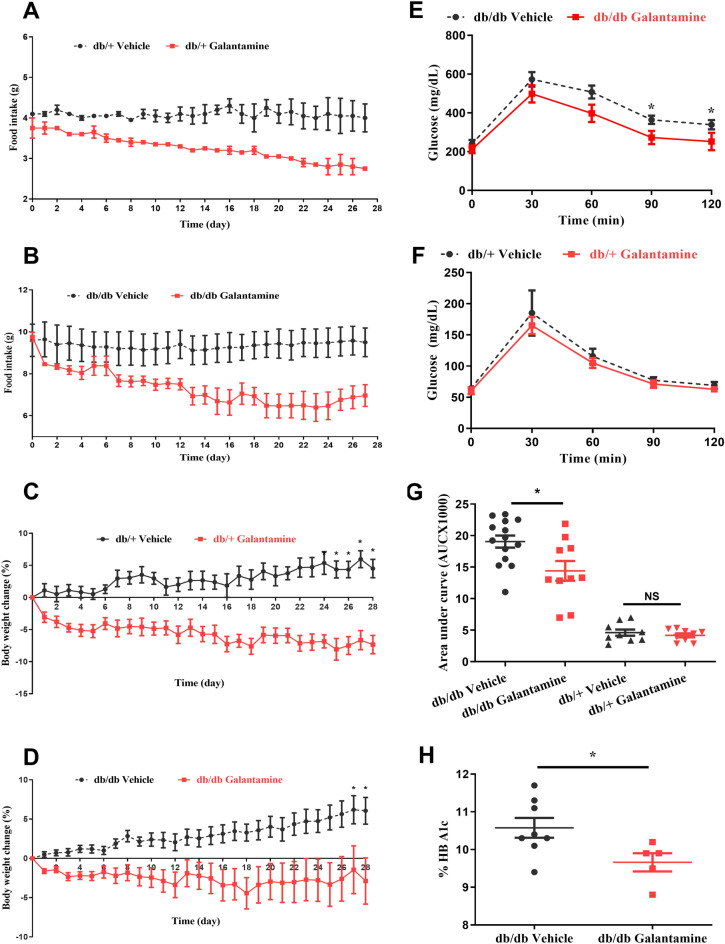
Effects of galantamine on food intake, body weight and glycemic control. All mice (7–8 weeks old) received galantamine (4 mg/kg, i.p., once daily) or vehicle for 4 weeks. Food intake and body weight were monitored daily. Food intake in db/+ mice in (**A**) and db/db mice in (**B**) were calculated daily. The changes in body weight (%) in db/+ mice in (**C**) and db/db mice in (**D**) were analyzed. The data is represented as mean ± SE (n = 5/group), *P < 0.05. OGTT was performed at week 4 in db/db mice in (**E**) and db/+ mice in (**F**) mice, as quantified by AUC analysis in (**G**) HbA1C in (**D**) was assayed using A1CNow^+^. The data is represented as mean ± SE (n = 5–12/group), *P < 0.05.

The ability of galantamine to alter food intake, body weight, and glycemic control was evaluated using db/+ and db/db mice (7–8 weeks old) because hyperphagia, weight gain and hyperglycemia are usually observed between 4 and 8 weeks of age^12,25^. Galantamine (4 mg/kg, i.p., once daily) or saline vehicle (i.p.) were administered daily for 4 weeks. Food intake (g) and body weight were measured daily. As shown in Fig. 1, daily food intake was higher in the db/db mice. Galantamine significantly reduced food intake (Fig. 1A,B) and body weight (Fig. 1C,D) in both the db+ and db/db mice compared to mice administered saline vehicle alone.

To examine the effects of galantamine on glycemic control, db/+ and db/db mice (7–8 weeks old) were treated with galantamine (4 mg/kg, i.p., daily) or saline vehicle for 4 weeks. An oral glucose tolerance test was performed (OGTT) as described in “Methods”. The OGTT area-under-the-curve (AUC) and glycosylated hemoglobin (HbA1c) were measured on day 28. No significant changes in glucose tolerance were found in galantamine-treated db/+ mice (Fig. 1E,F, NS P > 0.05). In contrast, the galantamine-treated db/db mice only demonstrate significant reductions in blood glucose at the 90- and 120-min time points after OGTT (Fig. 1F, *P < 0.05) and the AUC analysis demonstrates a 26% reduction in AUC values over the 120 min period (Fig. 1G, *P < 0.05). Consistent with this finding, the HbA1C level (Fig. 1H, *P < 0.05) was significantly reduced in galantamine-treated db/db mice.

**Figure 2:**
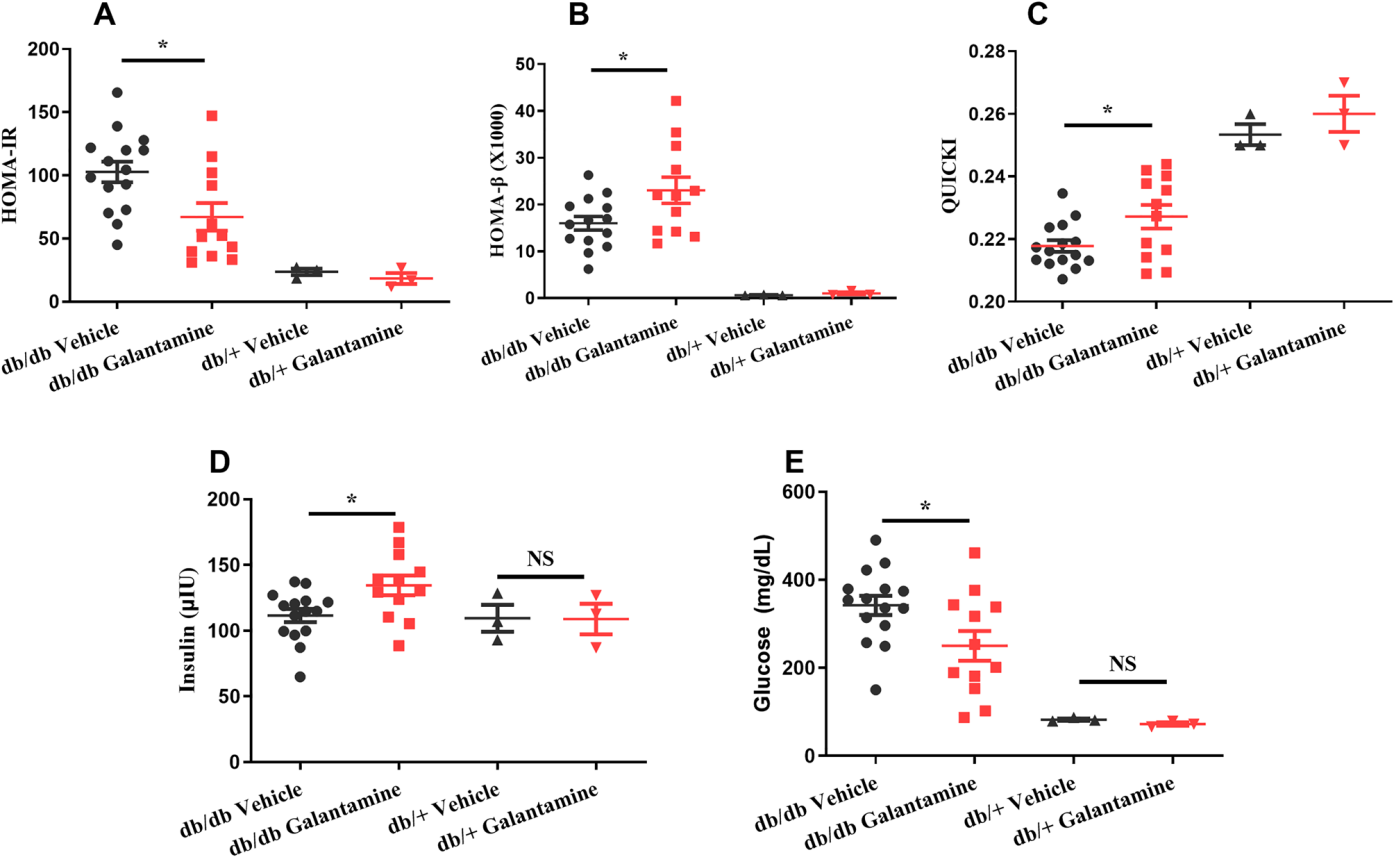
Effects of galantamine on insulin resistance and β-cell function. All mice received galantamine (4 mg/kg, i.p., once daily) or vehicle for 4 weeks. Glucose levels were checked in blood using glucometer. The plasma was collected for measuring insulin. HOMA-IR in (**A**), HOMA-β in (**B**) and QUICHI in (**C**) were calculated from the levels of glucose and insulin. The data is represented as mean ± SE (n = 3–15/group), *P < 0.05.

**Figure 3:**
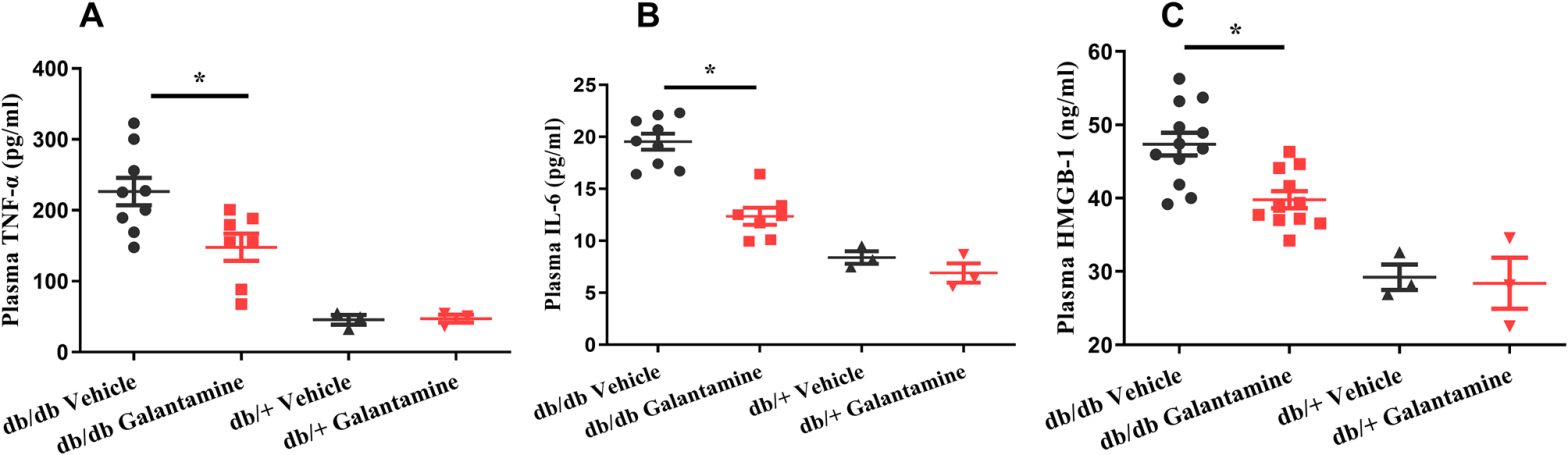
Effects of galantamine on inflammatory markers (TNF-α, IL-6 and HMGB-1). All mice (7–8 weeks old) received galantamine (4 mg/kg, i.p., once daily) or vehicle for 4 weeks. The plasma was collected for measuring plasma TNF-α in (**A**), IL-6 in (**B**), and HMGB-1 in (**C**) using ELISA. The data is represented as mean ± SE (n = 3–9/group), *P < 0.05.

Next, we used the homeostatic model assessment^26^ and quantitative insulin-sensitivity check index (QUICKI) to investigate the effects of galantamine on insulin resistance (HOMA-IR, QUICKI) and β-cell function (HOMA-β). Although these parameters were originally developed to assess insulin sensitivity and β-cell function in humans. They are commonly used to compare insulin resistance and β-cell function between groups of animals when assessing therapies to treat T2DM. Heterozygous db/+ and homozygous db/db mice were treated with galantamine (4 mg/kg, i.p., daily) or saline for 4 weeks. Fasting blood glucose and insulin were measured and HOMA-IR (Fig. 2A), HOMA-β (Fig. 2B), and QUICKI (Fig. 2C) were calculated as described in “Methods”. Reductions in HOMA-IR, an increase in HOMA-β and an increase in QUICKI were noted in the galantamine-treated db/db mice. A significant increase in insulin and reduction in glucose were observed in galantamine-treated db/db mice. (Fig. 2D,E). Collectively these findings suggest galantamine can lead to improvements in insulin sensitivity and β-cell function in the db/db mouse model. Levels of TNF-α (Fig. 3A), IL-6 (Fig. 3B) and HMGB1 (Fig. 3C) were elevated in db/db mice (*P < 0.05 vs. db/+) and decreased by galantamine (*P < 0.05 vs. db/db vehicle).

### Galantamine improves DN in db/db mice

The db/db mouse develops obesity by 4 weeks followed by hyperglycemia with glucosuria and albuminuria by 8 weeks of age. In db/db mice, DN is characterized by albuminuria, podocyte loss, and mesangial matrix expansion and increase in glomerular size^12,13,27^. Elevations in plasma BUN and creatinine are also seen. Histologic evidence of DN develops from 8 to 16 weeks including glomerular hypertrophy and mesangial matrix expansion. Increases in the extracellular matrix proteins type IV collagen and fibronectin, nodular glomerulosclerosis, and tubulointerstitial injury (tubular atrophy, dilatation, apoptosis) are seen by 4 months^12,13^. Homozygous db/db mice aged 16–17 weeks were used for the study of DN. Blood samples were collected at weeks 1 and 8 from mice given galantamine or saline for 8 weeks (4 mg/kg, i.p., once daily), while urinary samples were collected weekly for 8 weeks. After 8 weeks of treatment plasma and kidneys were analyzed for renal function, histology, apoptosis, SGLT-2 expression, activation of P38 MAPK signaling and caspase-1 activation.

**Figure 4:**
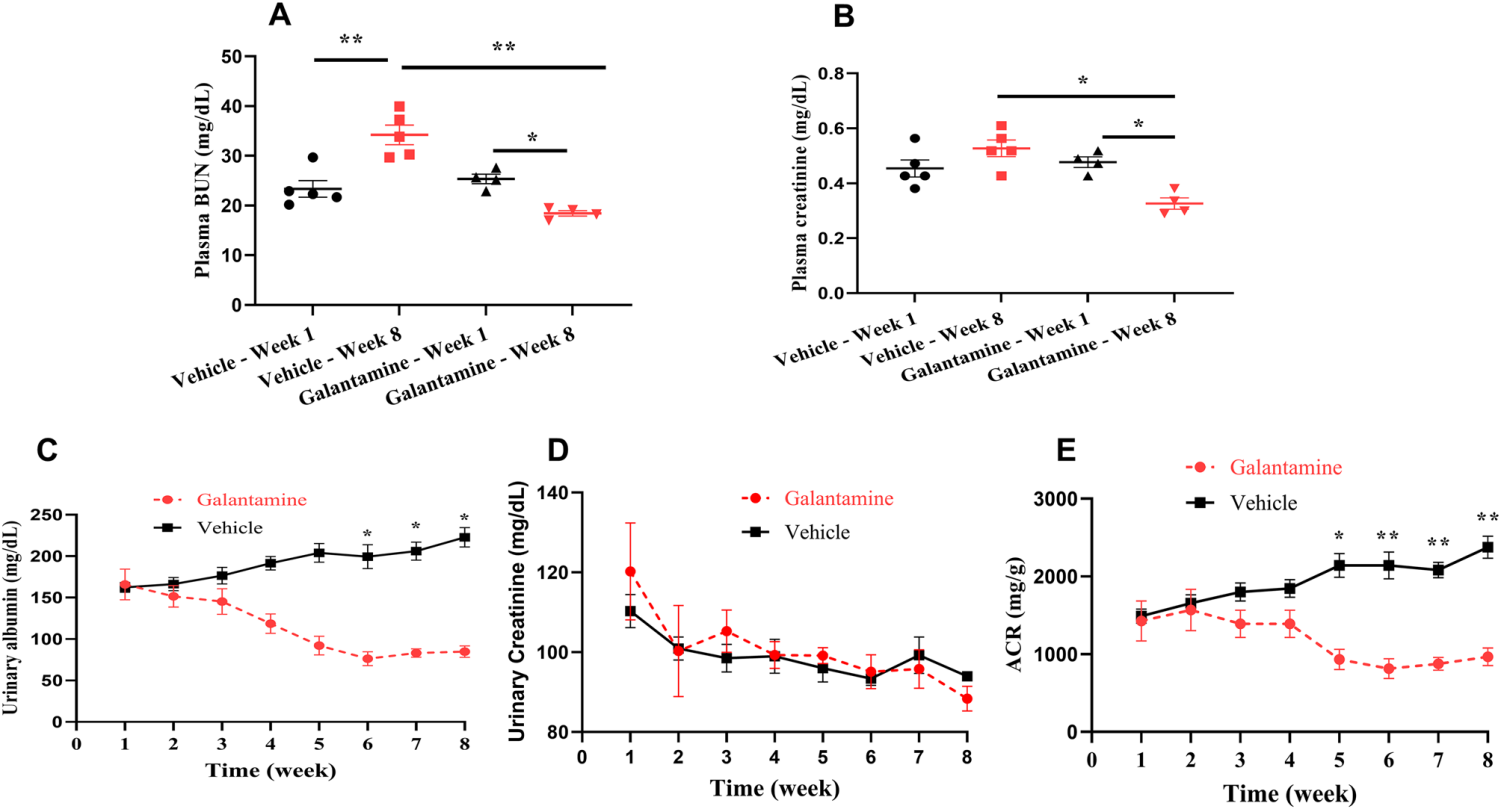
Effects of galantamine on renal function and injury. All mice (12–13 weeks old) received galantamine (4 mg/kg, i.p., once daily) or vehicle for 8 weeks. Blood samples were collected at weeks 1 and 8 for measuring plasma BUN in (**A**) and creatinine in (**B**). Urinary samples were collected weekly for 8 weeks for measuring urinary albumin in (**C**), creatinine in (**D**). ACR in (**E**) was calculate using urinary albumin and creatinine. The data is represented as mean ± SE (n = 3–5/group), *P < 0.05, **P < 0.01.

**Figure 5:**
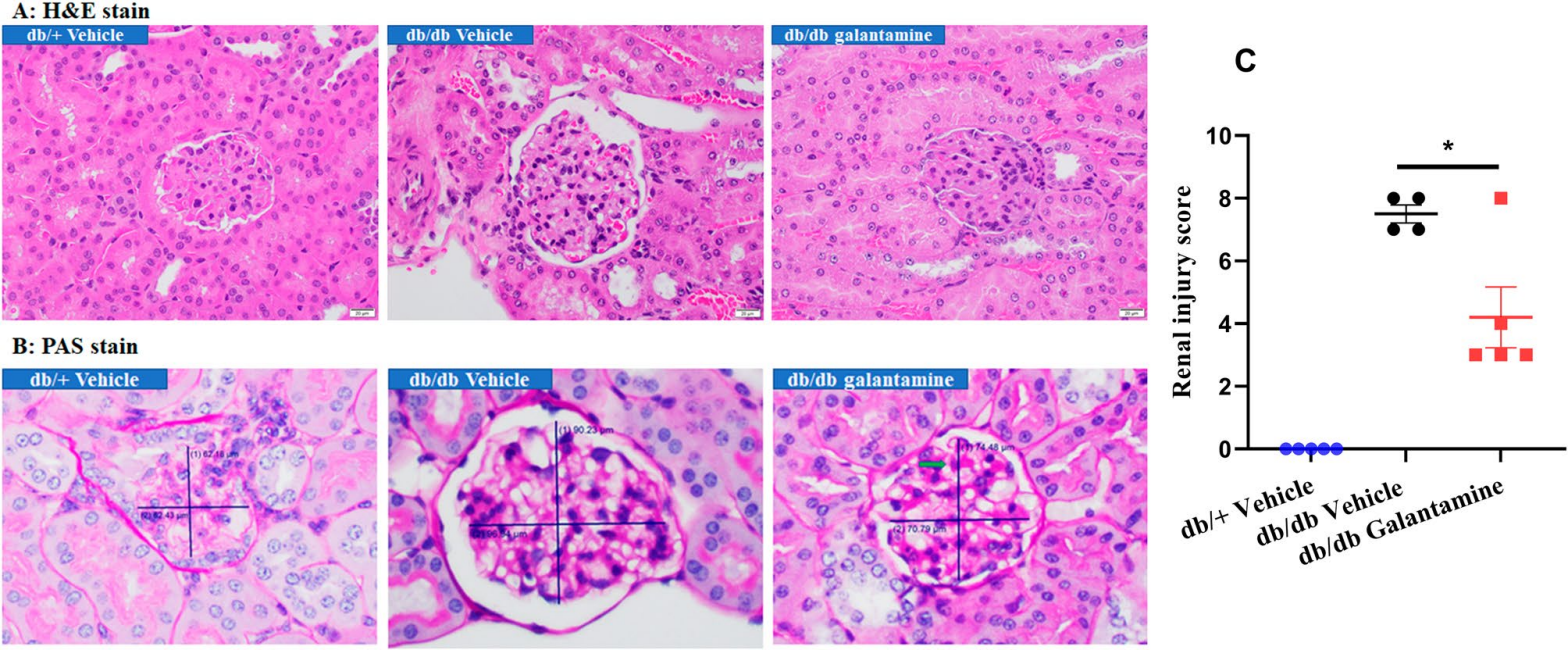
Histological assessment of DN. All mice (12–13 weeks old) were treated with galantamine (4 mg/kg, i.p., once daily) or vehicle for 8 weeks. Kidney sections were stained with H&E and PAS to assess the glomeruli and mesangial matrix, and detect acute pyelonephritis and ATN. Representative histological sections of the kidney are shown with H&E stain in (**A**) and PAS stain in (**B**). Magnification 400 ×. Histological changes in DN were characterized by glomerular diameter, acute pyelonephritis, ATN, percentage of glomeruli with nodular formulation and glomeruli mesangial expansion (green arrow). Quantification of renal injury was performed as described in “Methods” is shown in (**C**). The data is represented as mean ± SE (n = 4–6/group), *P < 0.05.

Vehicle-treated db/db mice demonstrate a significant increase in plasma BUN (Fig. 5A) by week 8 (**P < 0.01, week 1 vs. week 8). In contrast, galantamine-treated db/db mice demonstrate reductions in plasma BUN (Fig. 4A) and creatinine (Fig. 4B) (*P < 0.05, week 1 vs. week 8). Compared with vehicle-treated controls, the galantamine-treated db/db mice demonstrate improvements in plasma BUN and creatinine at 8 weeks (P < 0.05, vehicle vs. galantamine). Consistent with this finding, albuminuria in the galantamine-treated db/db mice is markedly improved (Fig. 4C). Urinary creatinine levels were similar between the groups (Fig. 4D), whereas the calculated albumin to creatinine ratio (ACR, Fig. 4E) was increased in the saline treated db/db mice compare to the galantamine-treated group (*P < 0.05, vehicle vs. galantamine). Collectively, these results provide evidence galantamine attenuates the development of DN in the db/db mouse model.

Next, we examined the effects of galantamine on renal histology. Kidneys were harvested from db/+ mice and from db/db mice treated with saline or galantamine for 8 weeks. H&E (Fig. 5A) or PAS (Fig. 5B) stained sections were assessed by a renal pathologist blinded to the experimental group. Characteristic histologic changes of DN (glomerular diameter, mesangial matrix expansion, nodular glomerulosclerosis, acute pyelonephritis and acute tubular necrosis) were quantified using the scoring system in Table 1. Compared with db/+ mice, the db/db mice showed signs of significant chronic glomerular injury: moderate mesangial matrix expansion with nodular formation and glomerular hypertrophy (glomerular diameter of 95 μm in db/db mice vs. 75 μm in db/+ mice). In addition, db/db mice showed more active tubular injury with inflammation (clusters of neutrophils and lymphocytes in the renal pelvis and cortical tubulointerstitium shown in supplemental file: s1.file). Administration of galantamine for 8 weeks reduced the histologic changes (glomerular hypertrophy, mesangial matrix expansion, tubular injury) and renal impairment scores in db/db mice, an effect that was significant in comparison to the saline control (Fig. 5C, *P < 0.05). These findings further confirm our hypothesis that galantamine improves histologic evidence of DN in db/db mice.

**Table 1:** Diabetic nephropathy histological scoring system.

Point	Glomerular diameter	Acute pyelonephritis	ATN (%)	Percentage of glomeruli with nodular formation (%)	Glomeruli mesangial expansion
0	< 80	No	< 5	1–10	No
1	80–100	Yes	5–15	11–25	Yes
2	> 100		16–25	> 25	
3			> 25		

**Figure 6:**
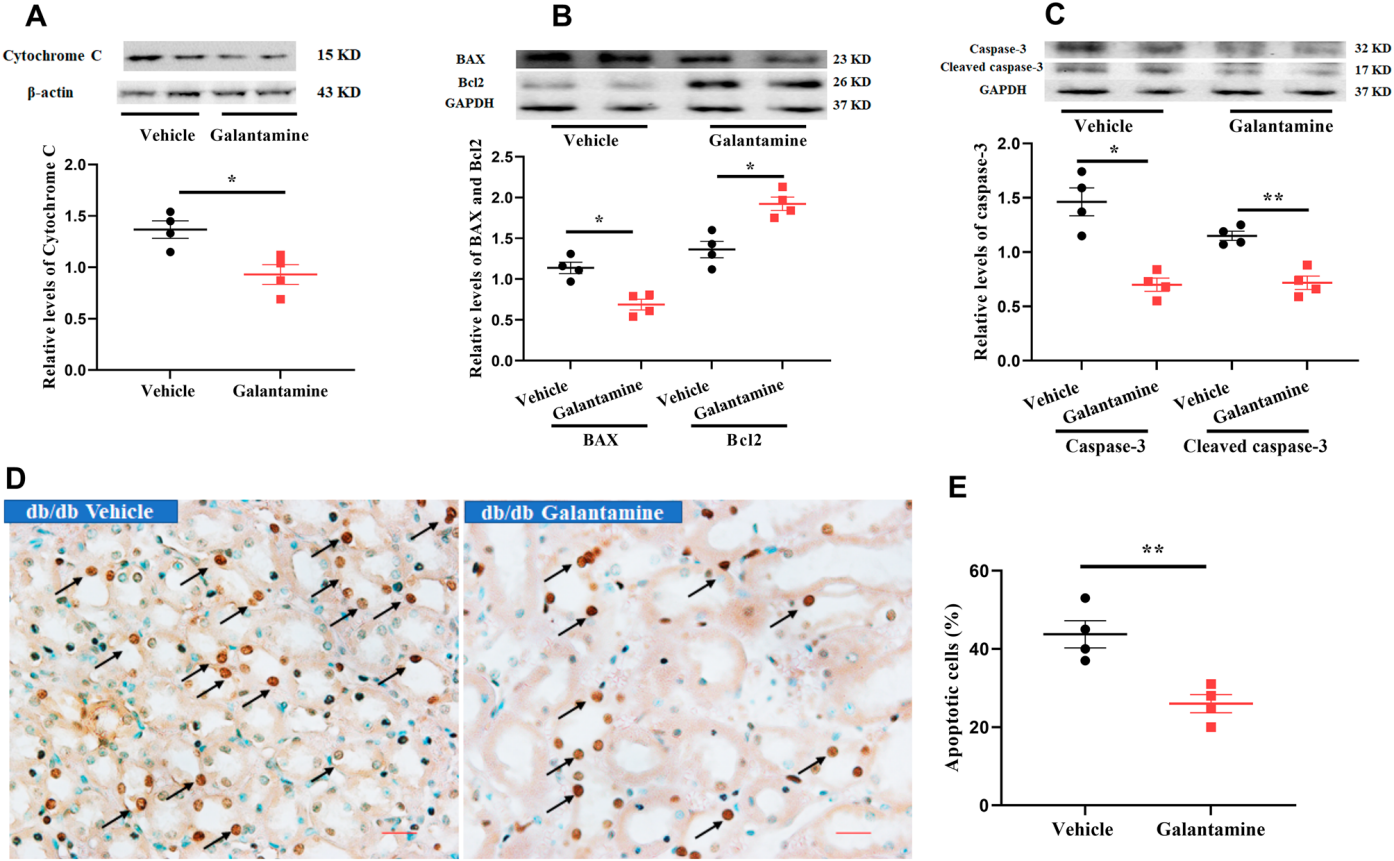
Galantamine attenuates mitochondrial dysfunction and renal apoptosis. The db/db mice (12–13 weeks old) were treated with galantamine (4 mg/kg, i.p., once daily) or vehicle for 8 weeks. Western blot was used to measure apoptotic proteins (cytochrome *c* in (**A**), BAX and Bcl2 in (**B**), and caspase-3 in (**C**). Densitometry data for the individual proteins was normalized to β-actin and GAPDH and is presented as relative densitometry units (RDU). Histological sections were stained using TUNEL assay to detect apoptotic cells in (**D**). Magnification 400 ×. Immunofluorescence stain shows TUNEL-positive cells (dark brown, black arrow), nuclei (blue). Quantification of TUNEL-positive cells was performed and represented in (**E**). The data is represented as mean ± SE (n = 4/group), *P < 0.05.

Apoptosis of tubular epithelial cells is a major feature of DN in the db/db mouse model which is triggered by the release of cytochrome *c* from damaged mitochondria. Galantamine-treated db/db mice demonstrated decreased levels of cytochrome *c* in the kidney (Fig. 6A) compared with vehicle treated mice (*P < 0.05, galantamine vs. vehicle). The apoptotic environment was assessed by measuring renal levels of pro-apoptotic proteins (BAX and cleaved caspase-3) and the anti-apoptotic protein Bcl2. Kidneys from db/db mice demonstrate a pro-apoptotic environment characterized by increased levels of Bax (Fig. 6B) and cleaved caspase-3 (Fig. 6C) with reductions in Bcl2 (Fig. 6B). All of these changes were significantly attenuated by galantamine. Consistent with this observation, the relative abundance of TUNEL-positive cells in kidneys from db/db mice were reduced by galantamine-treatment (Fig. 6D), with quantification of apoptotic cells shown in Fig. 6E (**P < 0.01, vehicle vs. galantamine).

### Circulating GLP-1 levels and renal SGLT-2 expression

**Figure 7:**
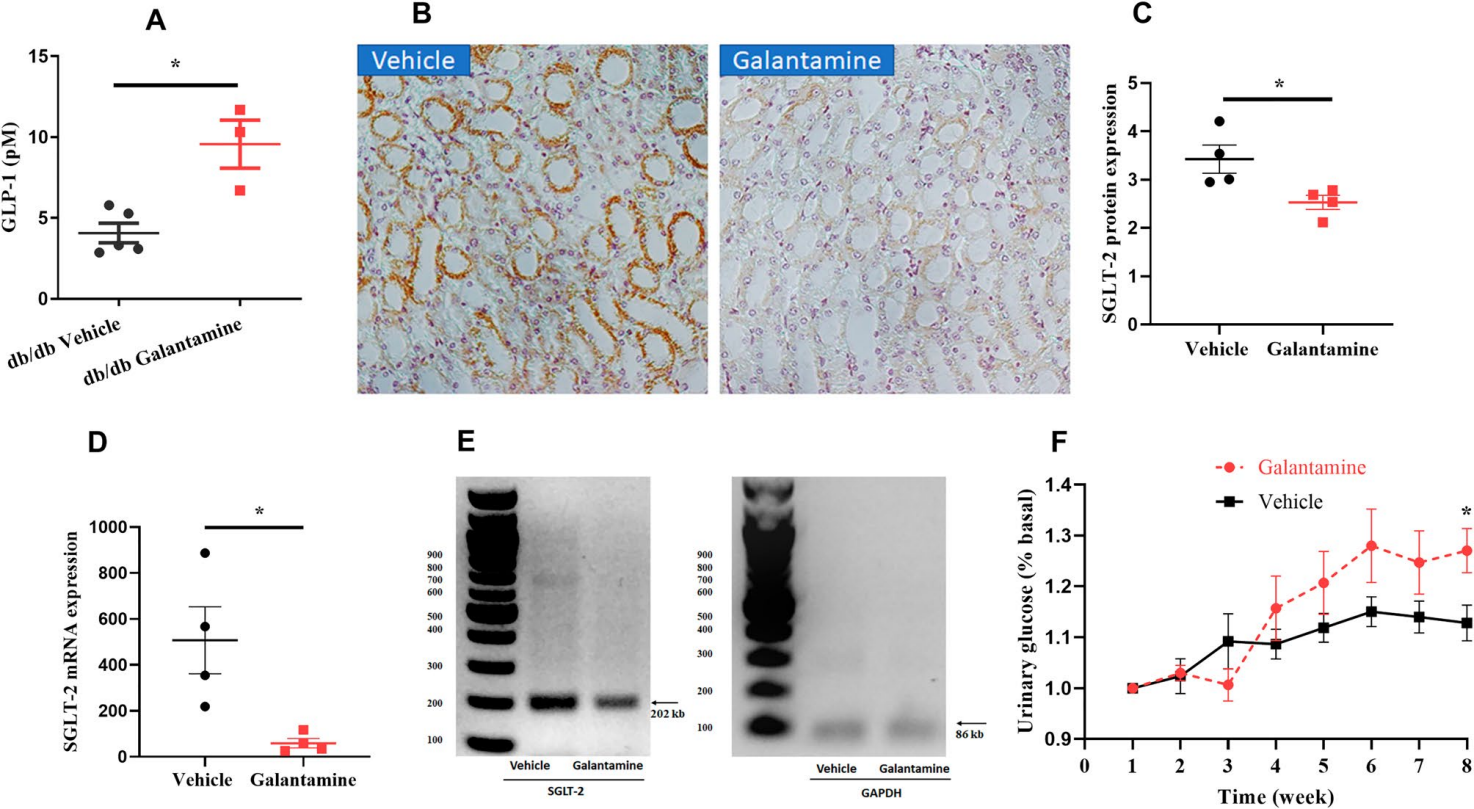
Renal expression of SGLT-2 and the levels of circulating GLP-1. Mice (12–13 weeks old) were treated with galantamine (4 mg/kg, i.p., once daily) or vehicle for 8 weeks. Plasma circulating GLP-1 levels were monitored in (**A**). Kidney sections were stained using immunohistochemistry to assess the expression of SGLT-2. Representative histological sections of the kidney are shown in (**B**) and quantification of renal SGLT-2 protein expression shown in (**C**). Renal SGLT-2 mRNA levels were detected by qRT-PCR and quantification of renal SGLT-2 mRNA expression shown in (**D**). PCR products (including SGLT-2 and GAPDH) were shown in (**E**) agarose gel. Urinary glucose levels in (**F**) were measured for 8 weeks. The data is represented as mean ± SE (n = 3–6/group), *P < 0.05.

To explore potential mechanisms by which galantamine improves glycemic control and DN we measured circulating glucagon-like peptide-1 (GLP-1) levels, renal Na^+^/glucose cotransporter-2 (SGLT-2) protein, mRNA expression and urinary glucose levels. GLP-1 is released by activation of the α7nAChRs on intestinal L cells and reduces postprandial elevations in blood glucose by multiple mechanisms^28^. SGLT-2 is a sodium-dependent glucose transporter found in renal proximal tubule (PTC) cells which reabsorbs up to 90% of filtered glucose in the kidney. Changes in the abundance or activity of renal SGLT-2 can significantly impact glycemic control by altering urinary glucose absorption. Galantamine administration significantly increased plasma levels of GLP-1 (Fig. 7A; *P < 0.05). In addition, galantamine-treated db/db mice also demonstrated decreased levels of SGLT-2 protein in renal tubules by immunohistochemistry (Fig. 7B,C, P < 0.05, galantamine vs. vehicle). As shown in Fig. 7D,E, the decrease in renal SGLT-2 protein in galantamine-treated mice was accompanied by a reduction in SGLT-2 mRNA (*P < 0.05, galantamine vs. vehicle) suggesting decreased SGLT-2 expression as a potential mechanism. Consistent with these observations, urinary glucose levels in the galantamine-treated mice increased over time and were significantly higher than saline treated mice by week 8 (Fig. 7F, *P < 0.05, galantamine vs. vehicle).

### Galantamine regulates renal p38 MAPK and caspase-1 activation in db/db mice

**Figure 8:**
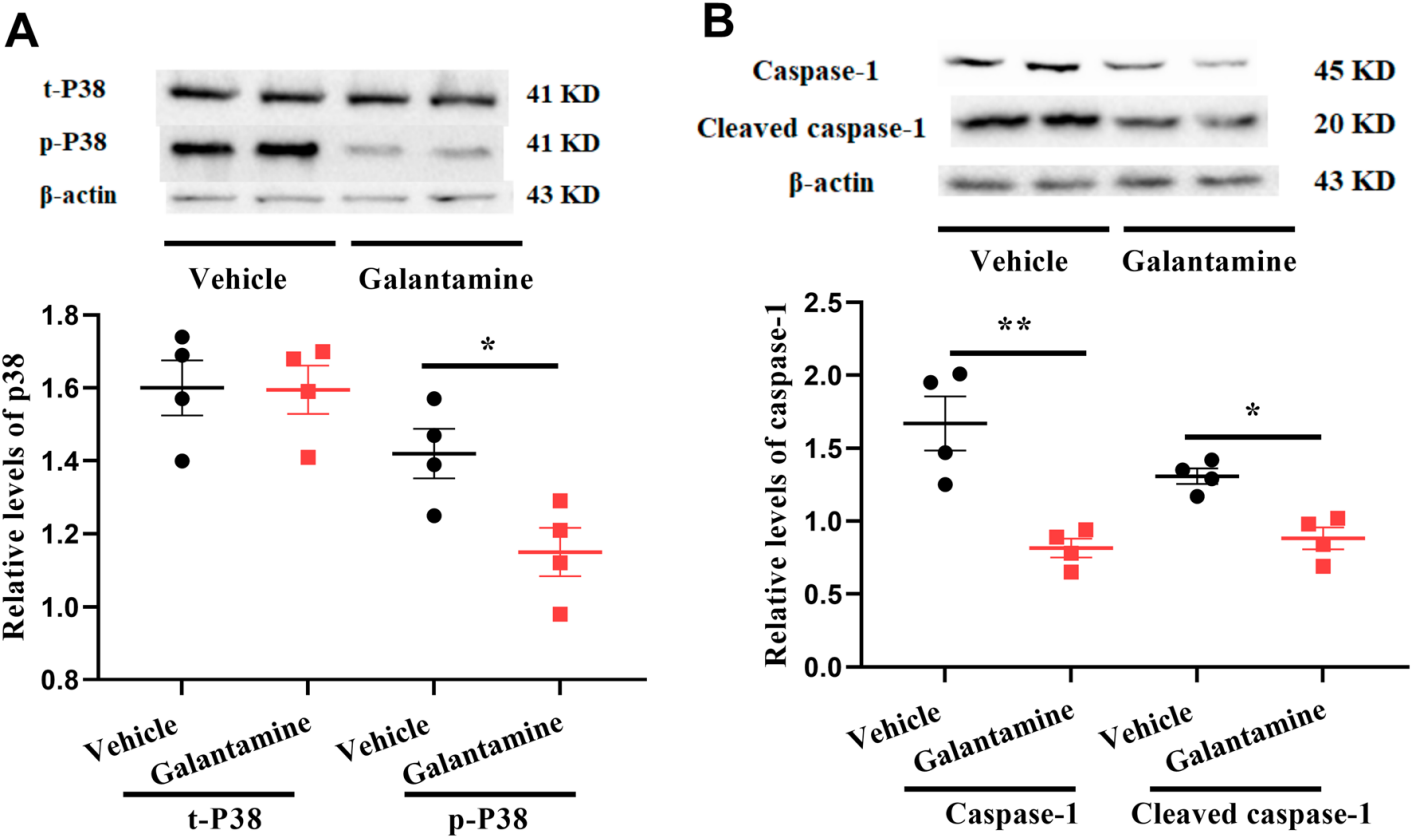
Activation of the caspase-1 and p38 MAPK pathways in db/db mice. Mice (12–13 weeks old) were treated with galantamine (4 mg/kg, i.p., once daily) or vehicle for 8 weeks. Renal lysate was prepared, then caspase-1 and p38 protein were assayed by Western blot. Densitometry data for phosphorylated and total and cleaved caspase-1 in (**A**), total and phosphorylated p38 in (**B**) were normalized to β-actin and presented as RDU. The data is represented as mean ± SE (n = 4/group), *NS* no significant difference, *P < 0.05.

Signal transduction is an essential biological process in cellular systems. Two signaling pathways which are important in regulating renal cell death are the p38 mitogen-activated protein kinase (p38 MAPK) and NLR Family Pyrin Domain Containing 3 (NLRP3) inflammasome caspase-1 signaling pathways. p38 MAPK can regulate apoptosis by affecting members of the Bcl-2 family of proteins including BAX and Bcl2. The NLRP3 inflammasome activates caspase-1, inducing cleavage and release of the proinflammatory cytokines and triggering pyroptosis^29^. Caspase-1 also has pro-apoptotic activity and helps activate caspase-3. To investigate the role of the p38 MAPK and caspase-1 signaling pathways in the db/db model of DN, phosphorylated p38 (p-P38 MAPK) and cleaved caspase-1 in kidney were measured by Western blot analysis. As shown in Fig. 8A,B, phosphorylated p38 and cleaved caspase-1 were decreased in kidney from db/db mice treated with galantamine (*P < 0.05, vehicle vs. galantamine). These results provide evidence that the p38 and caspase-1 signaling pathways are downregulated by galantamine in kidney from db/db mice.

## Discussion

The current study examines the effects of galantamine, a centrally acting acetylcholinesterase inhibitor approved for treating Alzheimer’s disease on glycemic control and DN in the db/db mouse model. The db/db mouse has a mutant leptin receptor which results in impaired satiety, hyperphagia and obesity which is commonly used to study T2DM and its complications. Our results provide evidence galantamine acts to improve glycemic control by reducing food intake, promoting weight loss, reducing systemic inflammation, improving insulin sensitivity, stimulating GLP-1 secretion, and decreasing renal SGLT-2 expression. While improvements in glycemic control undoubtedly help to prevent renal injury, galantamine also appears to attenuate renal apoptosis and inflammation, likely via its effects on the p38 MAPK and NLRP3 inflammasome/Caspase-1 pathways. Perhaps more interesting is the support our study provides for an important role of the CAP in the regulation of obesity, diabetes, and DN.

The effects of galantamine on food intake and weight loss in our study are supported by the results of others which show reductions of food intake and body weight in obese high-fat diet-fed mice^30^, the streptozotocin-induced (STZ) rat model of diabetes^31^, and in galantamine-treated patients with dementia^32^. Of interest, treatment with specific α7nAChR agonists like GTS-21 do not seem to impact food intake or body weight^33^. However, like galantamine, vagal nerve stimulation (VNS) has also been shown to suppress appetite, increase circulating GLP-1 and decrease inflammation^34–36^. The improvements in glycemic control and insulin resistance in our study are supported by similar findings in high-fat diet mice^30^ and the STZ rat model^31^. Galantamine was also shown to reduce inflammatory cytokine levels, insulin resistance and blood glucose levels in a double-blind clinical trial in patients with metabolic syndrome (ClinicalTrials.gov Identifier: NCT02283242)^37^. These changes were associated with alterations in autonomic tone suggesting involvement of the vagus nerve. Our results that galantamine treatment reduced inflammatory cytokines (TNF-α, IL-6, HMGB-1), improved insulin resistance and glycemic control in the db/db mice are consistent with those of the clinical trial of galantamine in patients with metabolic syndrome. Of interest, previous work from our laboratory showed that treatment of db/db mice with α7nAChR agonists (GTS-21 or PNU-282987) increased GLP-1 secretion and improved oral glucose tolerance by a mechanism requiring an intact GLP-1 receptor^21^. This suggests that some, but perhaps not all galantamine’s effects (e.g., decreased food and weight loss) on glycemic control and inflammation are mediated by the α7nAChR and GLP-1 receptor at the tissue level.

Galantamine likely improves glycemic control via multiple mechanisms including reduced food intake, body weight, increased circulating GLP-1 and decreased renal SGLT-2 expression. The relative importance of changes in food intake and body weight are difficult to ascertain since pair-feeding controls were not part of our study design. Endogenous GLP-1 regulates glycemia via its actions on the gastrointestinal tract, the vagus nerve, as well as pancreatic β cells (↑insulin synthesis, and secretion, ↓ apoptosis) and α cell (↓glucagon secretion). For a comprehensive review on GLP-1 regulation of glycemic control, see the article by Drucker^38^.

One of the more novel findings of our study are the reductions in renal SGLT-2 expression and glucosuria as a potential mechanism for improved glycemia in galantamine-treated db/db mice. Located on the luminal surface of the proximal renal tubule, SGLT-2 is a sodium-glucose cotransporter that is normally responsible for the reabsorption ~ 90% of the glucose filtered by the glomerulus^39^. SGLT-2 expression appears to be increased in the diabetic kidney and contributes to the development of hyperglycemia in T2DM. Consistent with this finding, SGLT-2 inhibitors (e.g., canagliflozin, empagliflozin) are highly effective in treating hyperglycemia in T2DM by enhancing glycosuria. Therefore, the reduction in renal SGLT-2 expression and increased glucosuria observed in galantamine-treated db/db mice likely contributed to the improvements on glycemic control we observed in our study.

Our results also show a beneficial effect of galantamine on renal function, albuminuria, histologic injury, and apoptosis in the db/db mouse model. Some of these findings are potentially attributable to the ability of galantamine to activate the α7nAChR since GTS-21 has also been shown to attenuate renal injury, histology, inflammation, and apoptosis in the db/db mouse model. However, galantamine’s effects on renal SGLT-2 represent another potential mechanism for its protective effects on DN in our study. SGLT-2 inhibitors have been shown to attenuate the development of DN in patients with T2DM. Several mechanisms have been identified for the beneficial effects of SGLT-2 inhibitors on DN including: (1) the restoration of tubuloglomerular feedback with reversal of afferent arteriole vasodilation and glomerular hyperperfusion; (2) a reduction in hyperglycemia-mediated reactive oxygen species (ROS); and (3) reduced renal tubular injury and inflammation^40,41^. Particularly interesting is the ability of SGLT-2 inhibitors to attenuate podocytopathy, elevate glomerular nephrin expression^42^ and alter glomerular distribution and size^15^ in db/db mice. Renal SGLT-2 expression and activity are regulated by filtered glucose levels, oxidative stress-induced inflammation, angiotensin II and its effects on epidermal growth factor activation^43^. Therefore, it is tempting to speculate that galantamine-mediated effects on glycemic control and inflammation are involved in the changes in renal SGLT-2 levels we observed in our study. Additional studies will be required to further delineate the relative importance of these SGLT-2 mediated mechanisms in the beneficial effects of galantamine on DN.

In the current study, down-regulation of renal apoptosis by galantamine was characterized by a reduction in the relative abundance of pro-apoptotic proteins (cytochrome *c*, BAX, and caspase-3) and increase in the anti-apoptotic protein (Bcl-2). There is evidence that SGLT-2 inhibitors provide an antioxidant mechanism of renoprotection^44–46^. However, our laboratory has also shown that α7nAChR activation by GTS-21 reduces apoptosis via regulating cytochrome *c*, BAX, Bcl-2, and caspase-3 in db/db mice^22^. Our current results showing a reduction of renal p38 MAPK activation by galantamine are consistent with Lim et al. who reported that knockout of the p38 MAPK signaling pathway in db/db mice protects against renal dysfunction, albuminuria, renal hypertrophy, loss of podocytes, activation of mesangial cells, and glomerular fibrosis^26^. The finding GTS-21 attenuates p38 MAPK activity and renal apoptosis in db/db mice is consistent with the effects of galantamine on p38 MAPK activity in the current study^22^. Our results also provide evidence galantamine attenuates renal caspase-1 activation by the NLRP3 inflammasome in the db/db model of DN. These findings are consistent with Shahzad et al. who demonstrate a protective effect of caspase inhibition and caspase-1 deficiency on DN in their study^47^. They also find that podocyte-specific caspase-1 genetic deficiency improves DN. Collective these studies support the idea that therapies like galantamine which enhance glycemic control, reduce renal inflammation, apoptosis, P38 MAPK and caspase-1 activation work through similar signaling pathways to prevent the progression of DN^48^.

Nonetheless, there are several limitations to our study. First, while the murine model of T2DM is useful experimentally, mutations in the leptin receptor are rare in humans. In addition, there are differences in insulin secretion and glucose metabolism between humans and mice, therefore our findings may not fully translate to the human condition. We also acknowledge that HOMA-IR and -B were designed and validated to study insulin sensitivity and β cell function in humans, despite this limitation they are commonly used to compare insulin resistance and β cell function in murine models of T2DM. Although we show galantamine ameliorates both glycemia and DN in the db/db model, the relative importance of vagal activation vs. α7nAChR activation and decreased renal SGLT-2 expression requires additional study. Finally, although we demonstrated galantamine improves both glycemia and DN in the db/db model, the pleiotropic effects of galantamine and our experimental design prohibit us from explicitly delineating the exact mechanisms for improvements in T2DM and DN.

In conclusion, our study provides evidence that galantamine acts to improve glycemic control in the db/db mouse by reducing food intake, enhancing weight loss, decreasing systemic inflammation, improving insulin sensitivity, stimulating GLP-1 secretion and decreasing renal SGLT-2 expression. Galantamine also attenuates the development of DN by improving glycemic control, reducing renal apoptosis and inflammation, in part through its effects on the 38 MAPK and NLRP3 inflammasome/Caspase-1 pathways. Collectively our data provide support for a potentially important role of the CAP in the regulation of obesity, diabetes and DN.

## Methods

### Animal models

Our studies with mice were approved by the Institutional Animal Care and Use Committee of SUNY Upstate Medical University (IACUC # 423). These studies were performed in accordance with the National Institutes of Health and ARRIVE guidelines for the use of laboratory animals. Heterozygous Lepr^db/+^ (db/+) mice (strain: BKS.Cg-*Dock7*^*m*^ +/+ *Lepr*^*db*^/J, Cat. No. 000642) were purchased from The Jackson Laboratory (Bar Harbor, ME). Homozygous Lepr^db/db^ (db/db) mice were obtained by breeding db/+ mice. Meanwhile, the obtained db/+ littermates as non-diabetes control were used to evaluate the effect of galantamine in diabetic and non-diabetic mice since db/+ mice do not exhibit the diabetic phenotype. Breeding of mice was performed at the Animal Core Facility at SUNY Upstate Medical University. Mice were housed in a temperature-controlled room at 22 °C. Mice aged 7–8 (for glycemic control study)^12^ and 16–17 (for DN study)^12,13^ weeks were randomly assigned to different treatment groups and matched for age and sex. Mice were housed in separate cages to measure daily food intake and body weight.

### Oral glucose tolerance test (OGTT)

All mice were fasted overnight prior to administration of glucose (2 g/kg body weight) by oral gavage. Tail blood samples were obtained before (time 0) and at 30, 60, 90 and 120 min after the glucose challenge for determination of blood glucose concentrations using an AimStrip Plus Blood Glucose Meter Kit (Cat. No. 37321, Germaine^®^ Laboratories, Inc, San Antonio, TX). The area under the curve (AUC) for OGTTs was calculated using the trapezoidal rule.

### Calculations of homoeostasis model assessment^26^ and the quantitative insulin sensitivity check index (QUICKI)

HOMA is a method for assessing beta-cell function (HOMA-β) and insulin resistance (HOMA-IR) from fasting glucose and insulin^49^. HOMA-β has been reported to correlate with β-cell function assessed by oral glucose tolerance test or hyperglycemic clamp test^50,51^. QUICKI is a useful index of insulin sensitivity and has linear correlation with glucose clamp determinations of insulin sensitivity^52^. The formulas used to calculate the three parameters are: HOMA-β = 360 × fasting insulin (μU/mL)/(fasting glucose (mg/dL) − 63). HOMA-IR = fasting glucose (mg/dL) × fasting insulin (μU/mL)/405. QUICKI = 1/[log (fasting insulin (μU/mL) + log (fasting glucose (mg/dL)].

### Galantamine drug delivery protocol and animal care

1. The effect of galantamine on glycemic control, food intake, body weight and inflammation in db/db and db/+ mice. Mice were divided into 4 groups at 7–8 weeks of age: (1) db/+ Vehicle, (2) db/+ Galantamine, db/db Vehicle, and (4) db/db Galantamine. Mice were then transferred to individual housing. These groups of mice were injected intraperitoneally (IP) once a day (Q.D.) for 4 weeks with the vehicle or Galantamine (4 mg/kg). Body weight and food intake were monitored daily and were calculated. OGTT was performed at week 4 of galantamine treatment. Blood was also collected at this time for the measurements of insulin, glucose, inflammatory mediator (HMGB-1) and HB Ac1.
2. The effect of galantamine on DN in db/db mice. Mice were divided into 2 groups at 12–13 weeks of age: (1) db/db Vehicle, and (2) db/db Galantamine. These groups of mice were injected intraperitoneally (IP) once a day (Q.D.) for 8 weeks with the vehicle or Galantamine (4 mg/kg). Body weight and food intake were monitored daily and were calculated. OGTT was performed at week 8 of galantamine treatment. Tail blood and urine were collected prior to the start of the experiment. Urine was collected weekly. At the end of the experiment (week 8), blood was collected and frozen at − 80 °C for subsequent analysis. Kidneys were collected and either flash-frozen in liquid nitrogen or fixed in 10% neutral formalin for histological analysis and immunohistochemistry.

### The measurements of kidney injury markers, HMGB-1 and HbA1c

Parameters from urine and blood are measured using the kits. Blood samples were centrifuged at 2500*g* for 15 min and plasma was collected for the measurements. Albumin (Cat. #: DIAP-25), creatinine (Cat. #: DICT-500) and blood urea nitrogen (BUN, Cat. #: DIUR-100) were assayed using the kits from BioAssay Systems. Total GLP-1 (Cat. #: EZGLP1T-36K, EMD Millipore) and HMGB-1 (Cat. #: MBS2021855, MyBioSource, Inc.) were measured by ELISA. HbA1c was measured using A1CNow^+^ (Ref #: 3021, PTS Diagnostics).

### Histological analysis

The H&E and Periodic Acid Schiff staining (Cat. #: ab150680, Abcam) of paraffin-embedded kidney sections (5 µm thick) was performed. Digital images from 20 glomeruli per animal were used at 400 × magnification under a microscope (Nikon, Melville, NY) to assess renal pathology alternations. A scoring system was established based on changes in the glomerular diameter, presence of acute pyelonephritis and/or acute tubular necrosis (ATN), percentage of glomeruli with nodular formation, and degree of glomeruli mesangial expansion (see Table 1 for detailed description).

### Immunocytochemistry

Paraffin sections were de-paraffinized in xylene and then hydrated in serial alcohol solutions. Tissue sections were incubated with 5% normal donkey serum and were either incubated for 1 h with SGLT-2 primary antibody (Cat. #: sc-393350, 1:50 dilution, SCBT) or overnight at 4 °C with α7nAchR. After three washes with PBS-T, sections were incubated with secondary antibody (Cat. #: sc-516102, 1:500 dilution, SCBT) for 1 h at room temperature. Sections were gently rinsed three times with PBS-T. Finally, ImmPACT DAB Peroxidase (HRP) Substrate (Cat. #: sk-4105, Vector) was added and samples were imaged with Nikon Eclipse TE 2000-U microscope (Nikon Instruments Inc., Melville, NY).

### Western blot analysis

Tissues were incubated with RIPA protein lysis buffer for 30 min at 4 °C, then centrifuged at 13,000 rpm for 5 min to sediment cell debris. Supernatants were collected for western blot analysis. Equal amounts (20 µg) of protein were loaded to each well, separated by SDS-PAGE, and then transferred to PVDF membrane (Cat. #: IPVH00010, Millipore Co., Ltd. USA). Membranes were blocked with 5% nonfat milk (Cat. #: MI17200, Research Products International) in Tris-buffered TBS plus 0.5% Tween-20 (Cat. #: BP337, Fisher Scientific) for 1 h at room temperature and incubated overnight at 4 °C with the indicated primary antibodies: total p38 (t-p38, 1:1000, Cat. #: 9212S), phospho-p38 (p-p38, 1:1000, Cat. #: 9211S) and Caspase-1 (1:1000, Cat. #: 24232T), cleaved caspase-1 (1:1000, Cat. #: 89332T) and caspase-3 (1:1000, Cat. #: 9662S) from Cell Signaling Technologies; caspase-3, (GGG), cleaved caspase-3 (1:1000, Cat. #: ab49822) from Abcam; Bcl-2 (1:200, Cat. #: sc-7382), Bax (1:200, Cat. #: sc-7480), GAPDH (1:2000, cat. #: sc-365062) and β-actin (1:4000, Cat. #: sc-47778) from Santa Cruz. After primary antibody incubation, the blot was incubated with HRP-conjugated secondary antibody for 1 h at room temperature. Antibody-antigen complexes were visualized with ECL (Cat. #: 34580, Thermo Scientific, IL) and analyzed quantitatively by densitometry with Image J software. The relative density of immunoreactive bands was normalized to the density of the corresponding β-actin and GAPDH.

### Measurement of mRNA levels by quantitative Real-time reverse transcription-PCR (qRT-PCR)

Total RNA was isolated from cells by the TRIzol method (Tri-Xtract, Cat. #: 214T-A, G-BIOSCIENCES). Total RNA (2 µg) was converted to cDNA using an iScript™ Reverse Transcription Supermix (Cat. #: 1708841, BIO-RAD). PCR was performed with the Step One Plus Real-Time PCR System with Step One software V2.0 (Applied Biosystems, Forster City, CA) according to the manufacturer’s guidelines. qRT-PCR was performed using SsoAdvanced™ Universal SYBR^®^ Green Supermix (Cat. #: 1725271, BIO-RAD) according to the manufacturer’s protocol. Relative gene expression was determined by the CT method, and mRNA levels were normalized to GAPDH. For PCR amplification, the specific primers used were SGLT-2 (Sense 5′-CCC ATC CCT CAG AAG CAT CTC C-3′ and antisense 5′-CTC ATC CCA CAG AAC CAA AGC A-3′) and GAPDH (Sense 5′-CAA TGT GTC CGT CGT GGA-3′ and antisense 5′-GAT GCC TGC TTC ACC ACC-3′. The PCR products from control and galantamine-treated mice were also run on an agarose gel and visualized using Gel imager (Axygen^®^ Gel Documentation Systems, Corning).

### Assessment of apoptotic cells

To detect renal apoptotic cells, we used the deoxynucleotidyl transferase-mediated dUTP nick-end labeling (TUNEL) kit (Cat. #: ab206386, Abcam) according to the manufacturer's instructions. Briefly, sections were deparaffinized with xylene, dehydrated to water through a graded alcohol series, and treated with permeabilization solution. The labeling reaction was performed using a solution containing terminal deoxynucleotidyl transferase. After staining, sections were mounted with DAPI (Cat. #: ab104139, Abcam) in a fluorescent shielding mounting medium to visualize cell nuclei. Apoptotic cells were quantified by counting TUNEL-positive cells from five randomly selected consecutive fields at 400 × magnification in a blinded manner by two experienced investigators. Apoptotic index was calculated as the number of TUNEL-positive cells expressed as a percentage of total cells.

### Statistical analysis

Data are presented as mean ± SEM (with Gaussian distribution) or median ± interquartile range (with no Gaussian distribution) analyzed using GraphPad Prism 9. The Mann–Whitney test or *t*-test was used to compare differences between two independent groups. Multiple group differences were determined using a one-way analysis of variance (ANOVA) with Bonferroni's multiple comparison test or a Kruskal–Wallis test with Dunn's multiple comparison test. A *p* value < 0.05 was considered significant. Data are from three or more independent experiments. Sample sizes (N) are shown in each figure legend.

### Supplementary Information

Supplementary Figures.

Supplementary Information.

## Data Availability

The original contributions presented in the study are included in the article/supplementary material. Further inquiries can be directed to the corresponding author.
