# Supplementary material for: Galantamine improves glycemic control and diabetic nephropathy in Leprdb/db mice
Source: Sci Rep. 2023 Sep 20;13:15544. doi: 10.1038/s41598-023-42665-2 (PMC10511534; doi:10.1038/s41598-023-42665-2)
Supplement: Supplementary file 1 — Supplementary Figures. [file 41598_2023_42665_MOESM1_ESM.pptx]

## Slide 1
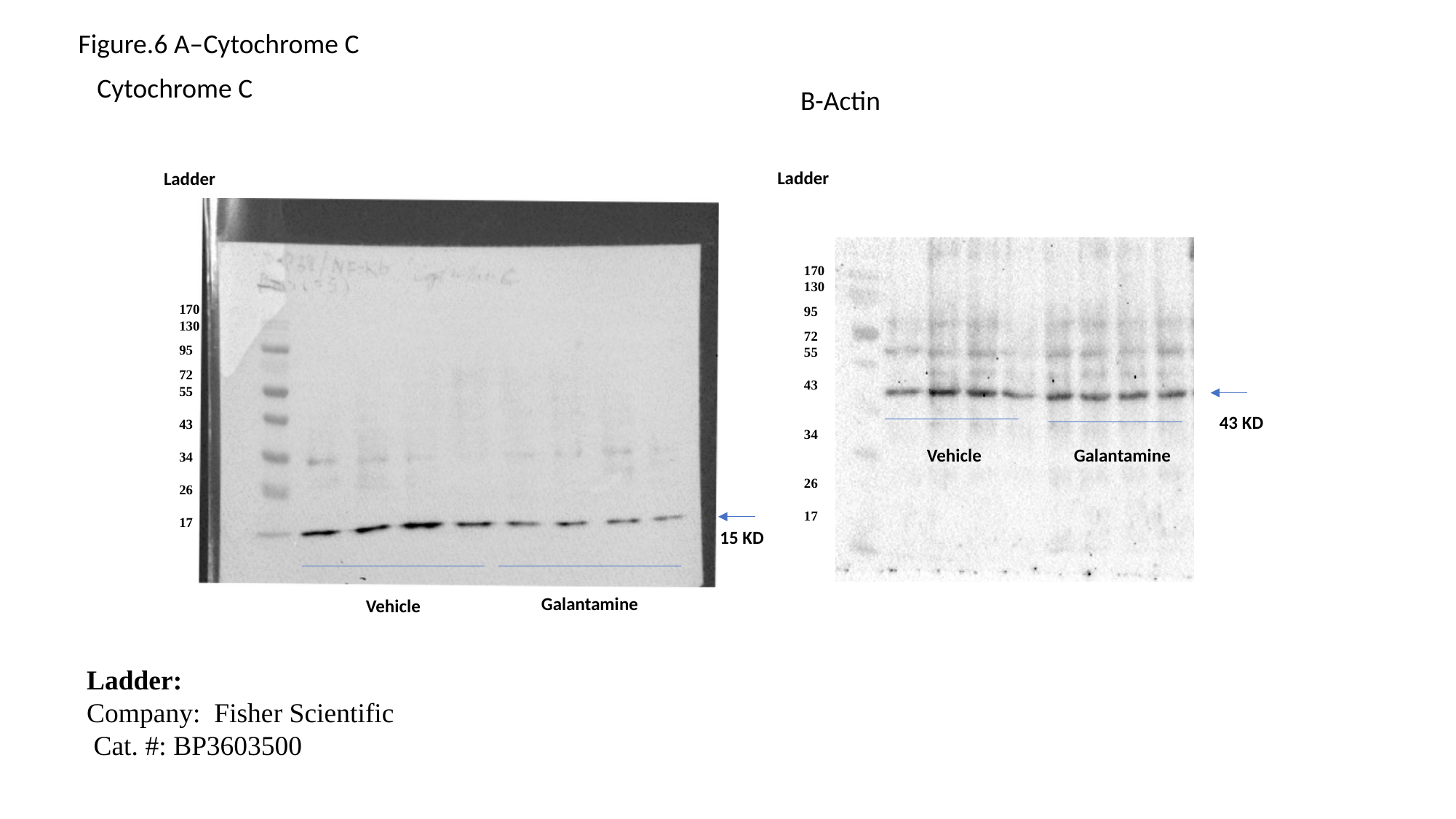

Figure.6 A–Cytochrome C
Cytochrome C
B-Actin
Ladder
Ladder
170
130
95
72
55
43
34
26
17
170
130
95
72
55
43
34
26
17
43 KD
Galantamine
Vehicle
15 KD
Galantamine
Vehicle
Ladder:
Company: Fisher Scientific
 Cat. #: BP3603500

## Slide 2
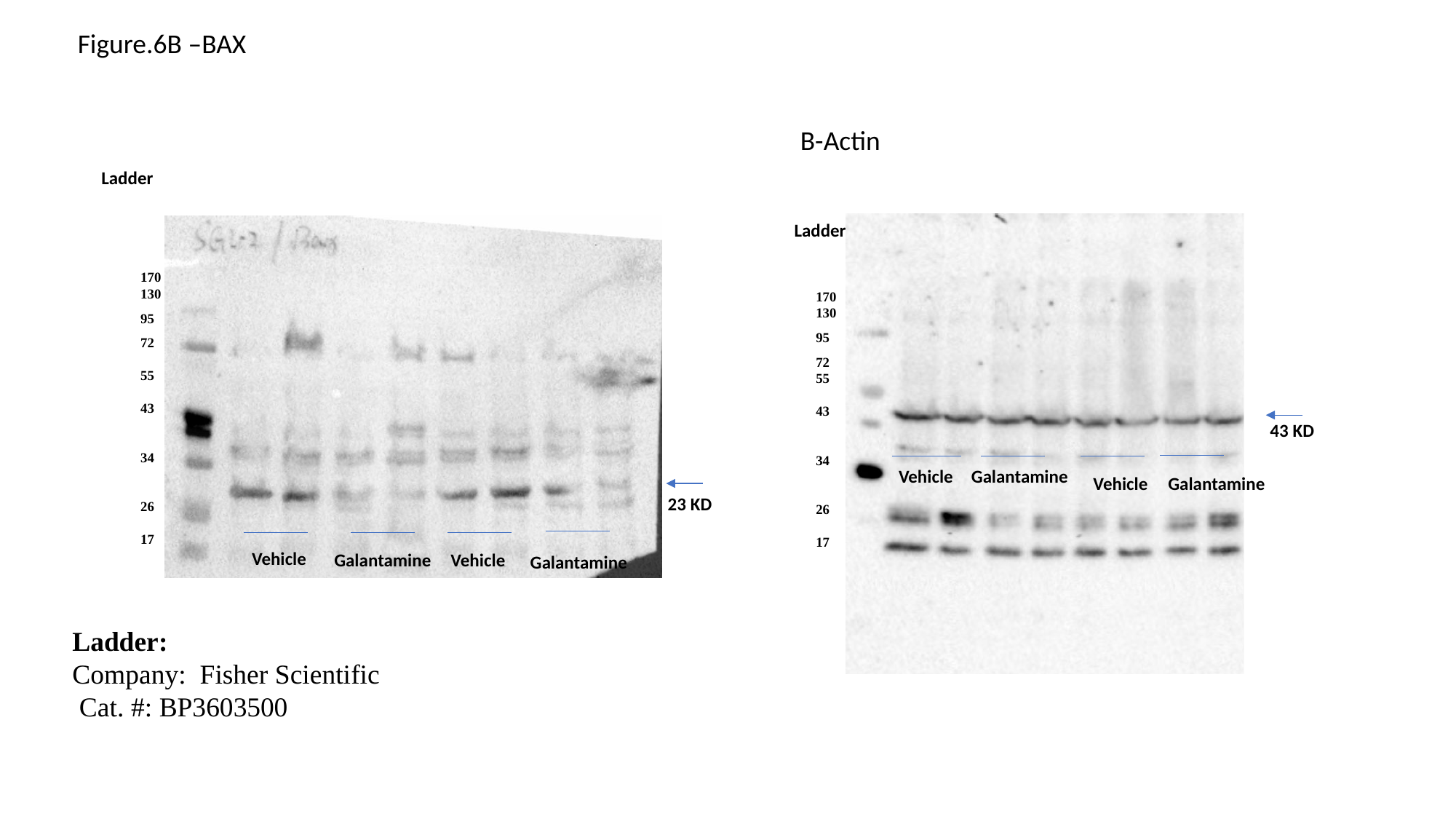

Figure.6B –BAX
B-Actin
Ladder
Ladder
170
130
95
72
55
43
34
26
17
170
130
95
72
55
43
34
26
17
43 KD
Vehicle
Galantamine
Vehicle
Galantamine
23 KD
Vehicle
Galantamine
Vehicle
Galantamine
Ladder:
Company: Fisher Scientific
 Cat. #: BP3603500

## Slide 3
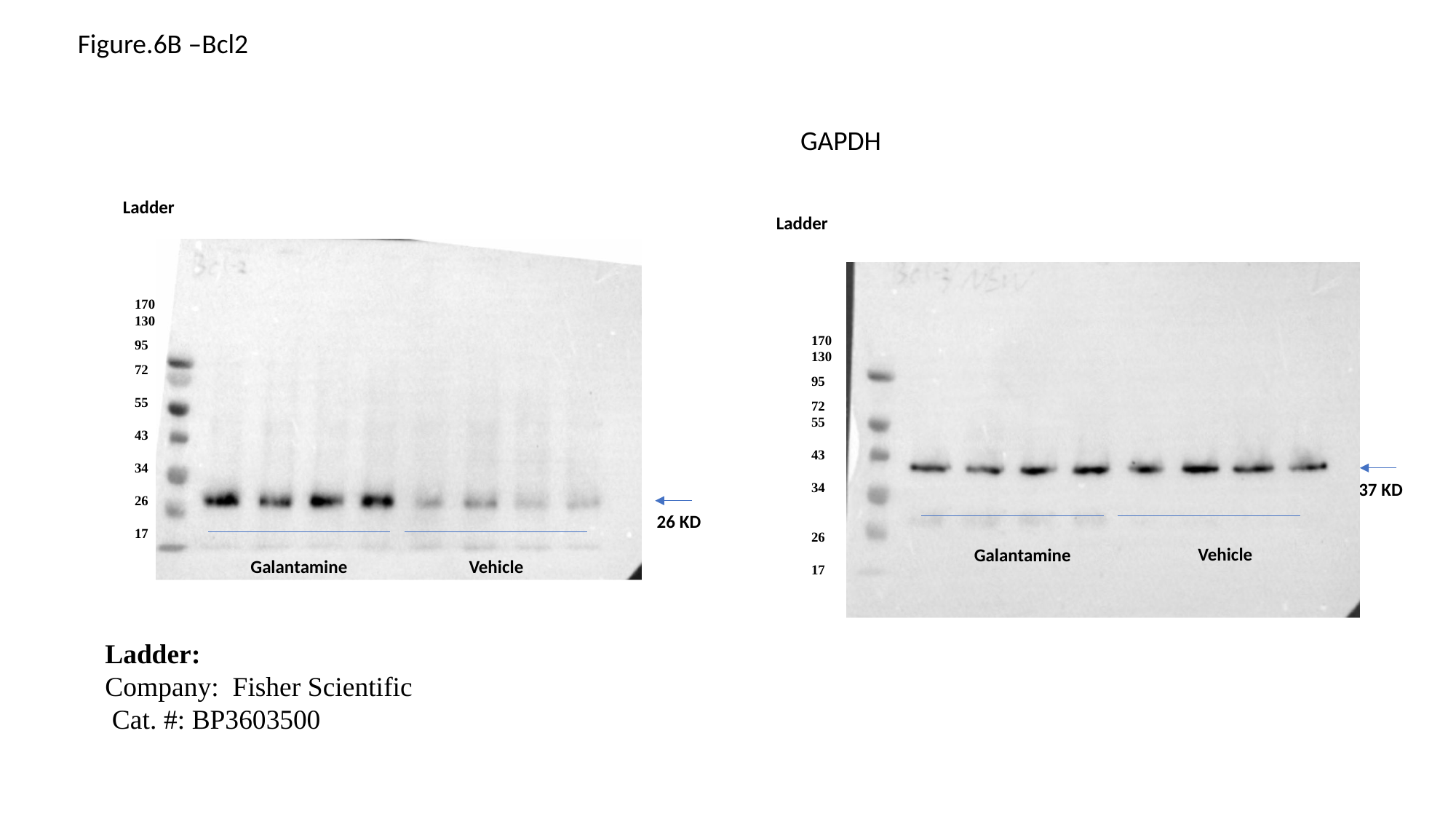

Figure.6B –Bcl2
GAPDH
Ladder
Ladder
170
130
95
72
55
43
34
26
17
170
130
95
72
55
43
34
26
17
37 KD
26 KD
Vehicle
Galantamine
Galantamine
Vehicle
Ladder:
Company: Fisher Scientific
 Cat. #: BP3603500

## Slide 4
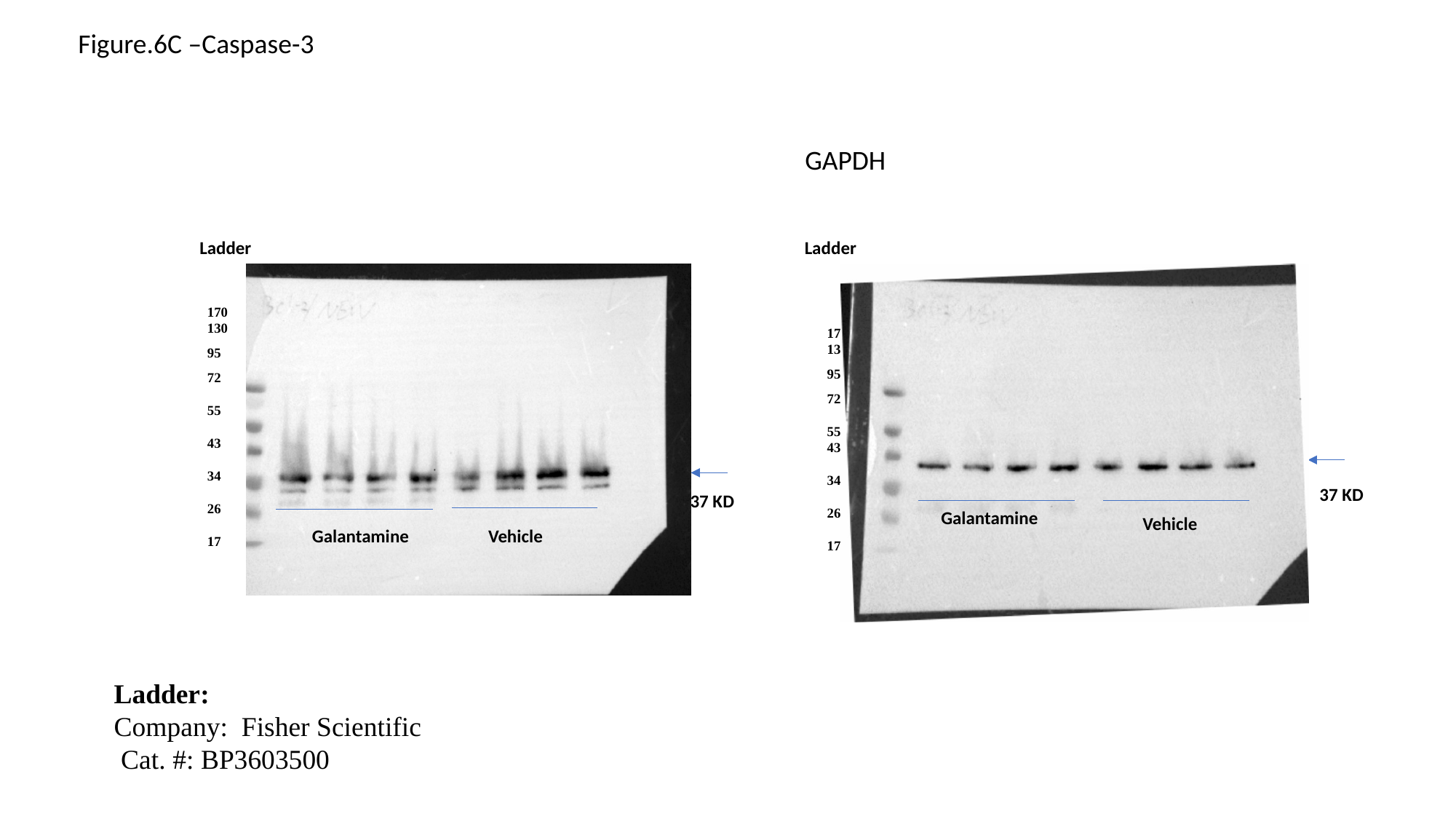

Figure.6C –Caspase-3
GAPDH
Ladder
Ladder
170
130
95
72
55
43
34
26
17
170
130
95
72
55
43
34
26
17
37 KD
37 KD
Galantamine
Vehicle
Galantamine
Vehicle
Ladder:
Company: Fisher Scientific
 Cat. #: BP3603500

## Slide 5
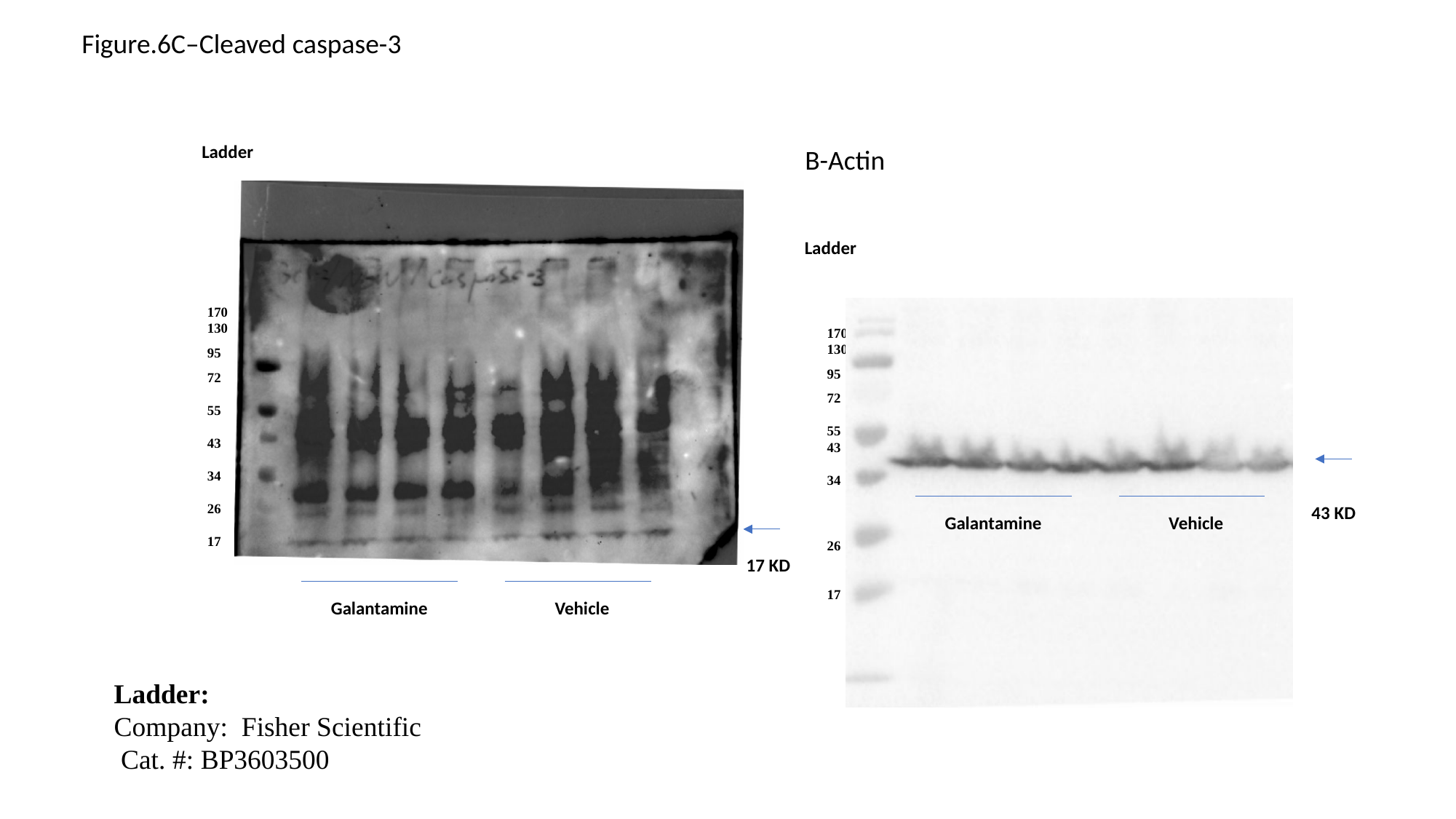

Figure.6C–Cleaved caspase-3
Ladder
B-Actin
Ladder
170
130
95
72
55
43
34
26
17
170
130
95
72
55
43
34
26
17
43 KD
Galantamine
Vehicle
17 KD
Galantamine
Vehicle
Ladder:
Company: Fisher Scientific
 Cat. #: BP3603500

## Slide 6
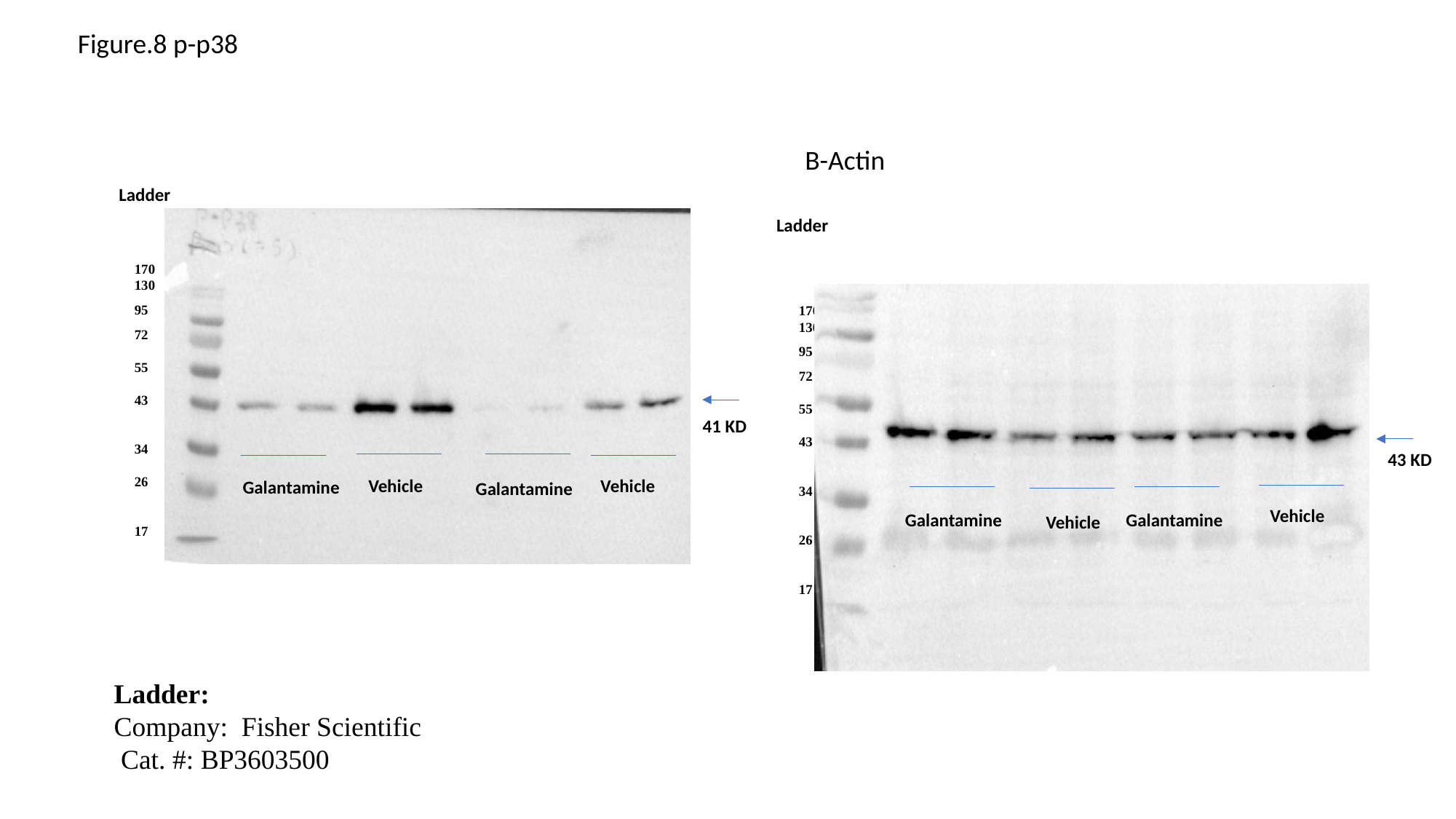

Figure.8 p-p38
B-Actin
Ladder
Ladder
170
130
95
72
55
43
34
26
17
170
130
95
72
55
43
34
26
17
41 KD
43 KD
Vehicle
Vehicle
Galantamine
Galantamine
Vehicle
Galantamine
Galantamine
Vehicle
Ladder:
Company: Fisher Scientific
 Cat. #: BP3603500

## Slide 7
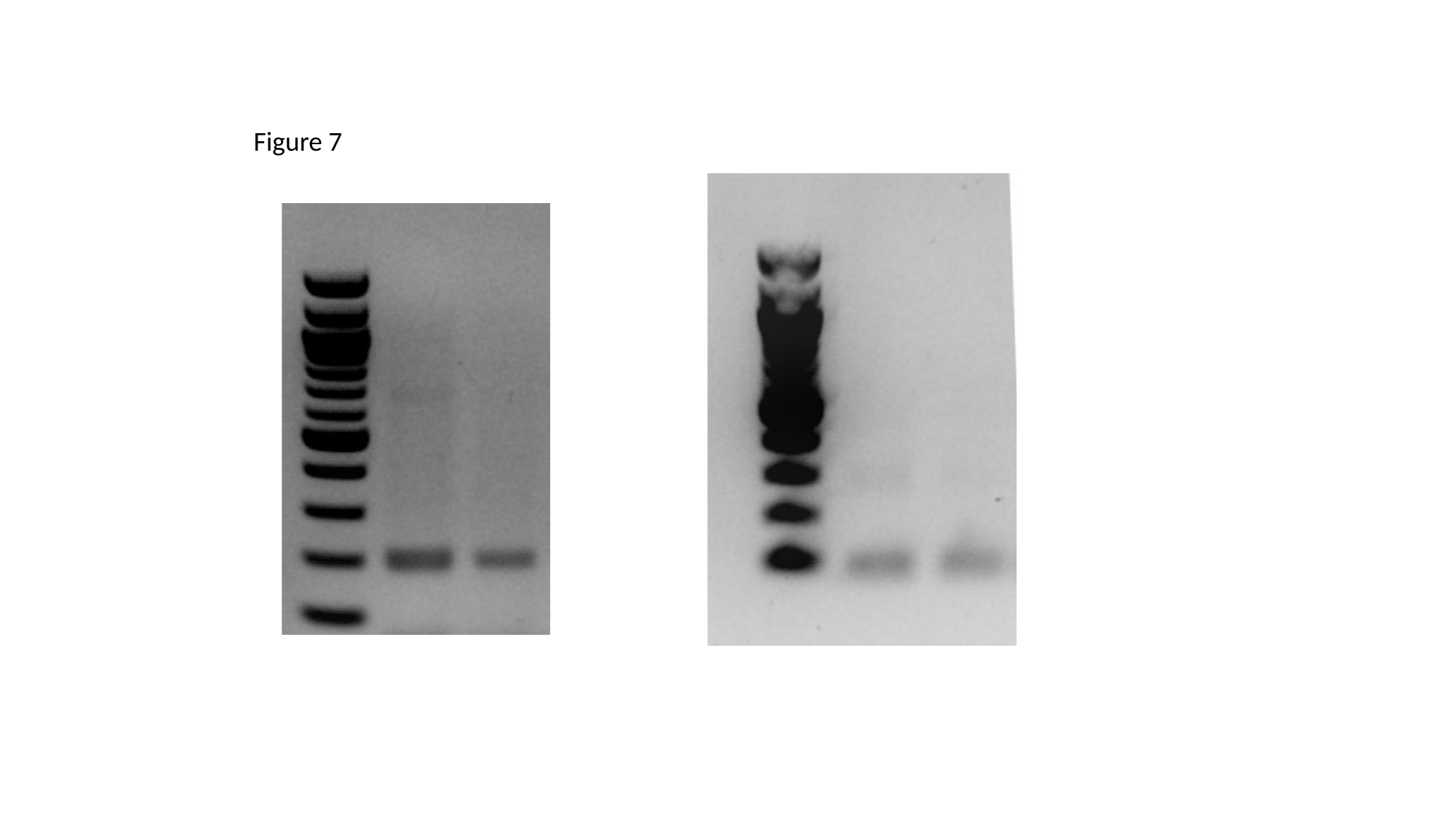

Figure 7

## Slide 8
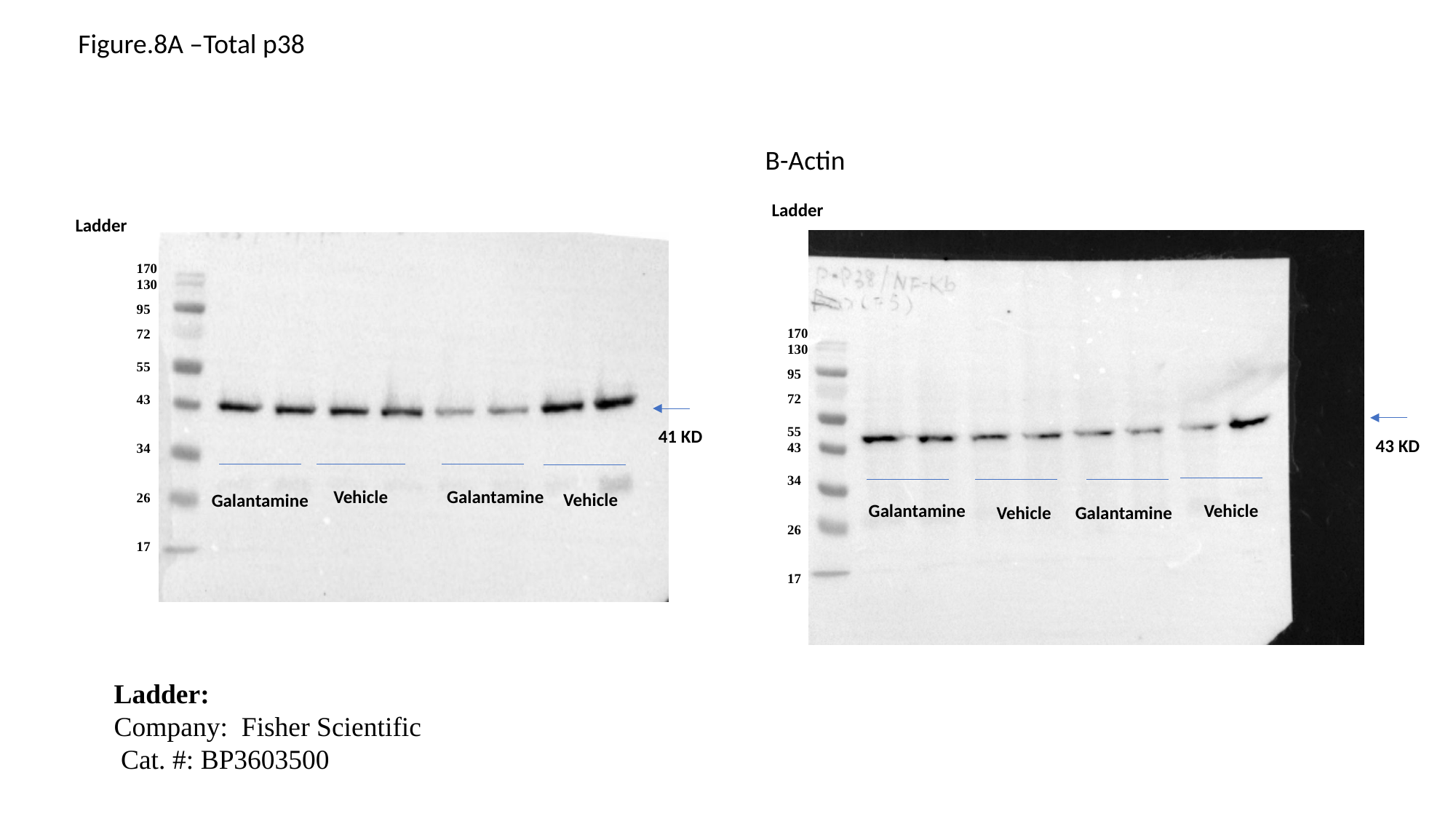

Figure.8A –Total p38
B-Actin
Ladder
Ladder
170
130
95
72
55
43
34
26
17
170
130
95
72
55
43
34
26
17
41 KD
43 KD
Vehicle
Galantamine
Vehicle
Galantamine
Galantamine
Vehicle
Vehicle
Galantamine
Ladder:
Company: Fisher Scientific
 Cat. #: BP3603500

## Slide 9
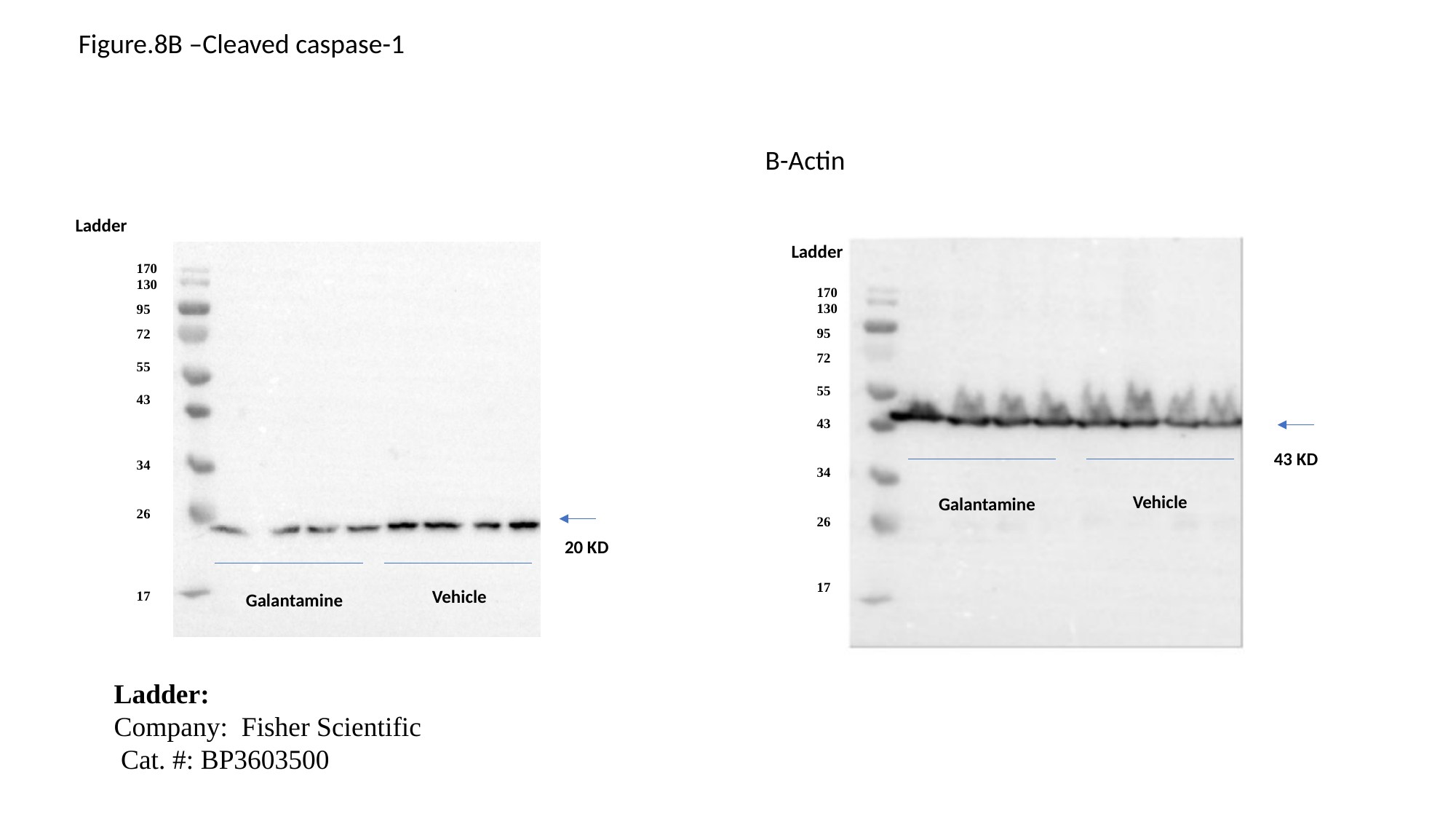

Figure.8B –Cleaved caspase-1
B-Actin
Ladder
Ladder
170
130
95
72
55
43
34
26
17
170
130
95
72
55
43
34
26
17
43 KD
Vehicle
Galantamine
20 KD
Vehicle
Galantamine
Ladder:
Company: Fisher Scientific
 Cat. #: BP3603500

## Slide 10
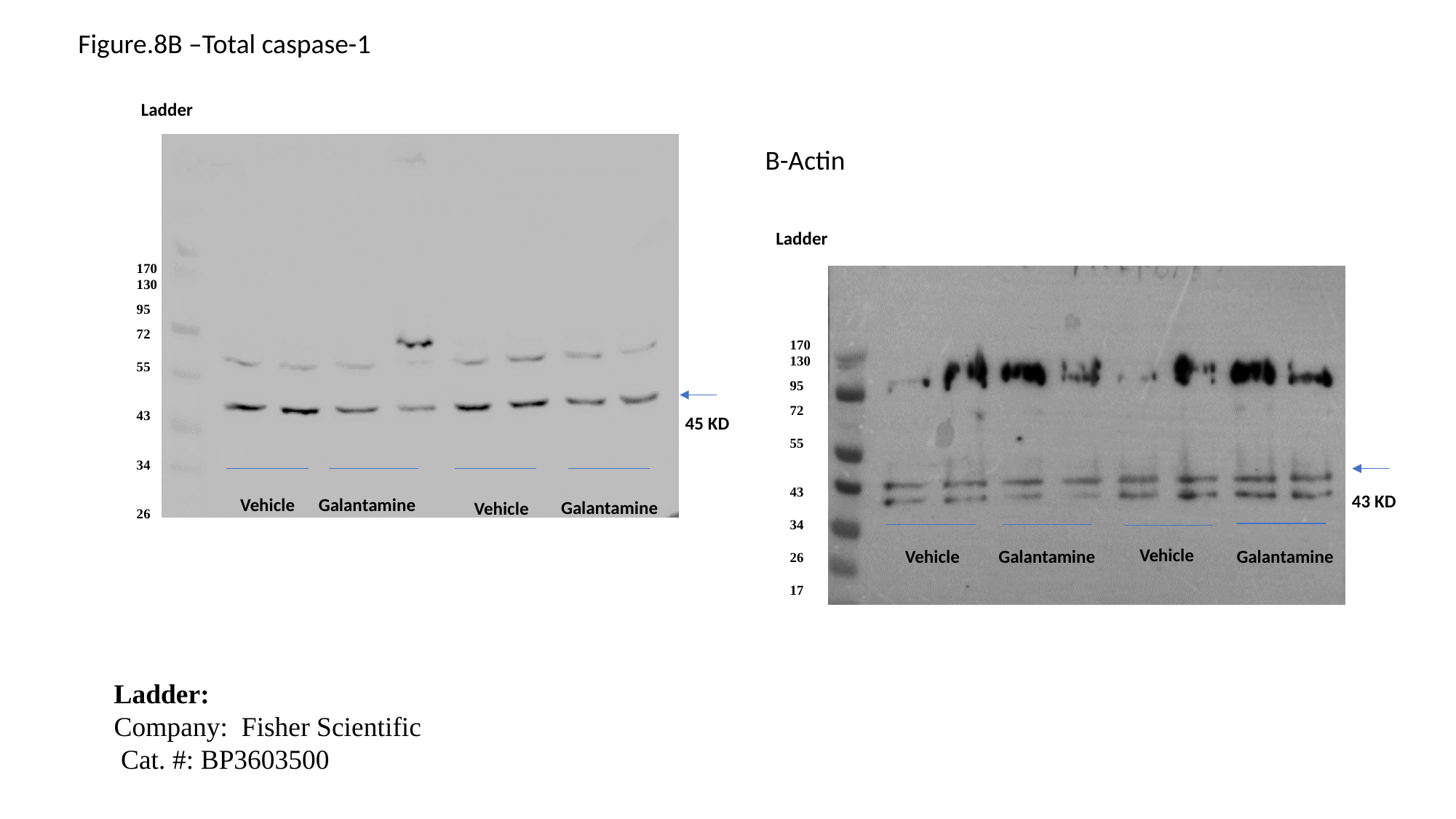

Figure.8B –Total caspase-1
Ladder
B-Actin
Ladder
170
130
95
72
55
43
34
26
170
130
95
72
55
43
34
26
17
45 KD
43 KD
Vehicle
Galantamine
Galantamine
Vehicle
Vehicle
Vehicle
Galantamine
Galantamine
Ladder:
Company: Fisher Scientific
 Cat. #: BP3603500
