# Supplementary material for: Galantamine improves glycemic control and diabetic nephropathy in Leprdb/db mice
Source: Sci Rep. 2023 Sep 20;13:15544. doi: 10.1038/s41598-023-42665-2 (PMC10511534; doi:10.1038/s41598-023-42665-2)
Supplement: Supplementary file 2 — Supplementary Information. [file 41598_2023_42665_MOESM2_ESM.pptx]

## Slide 1
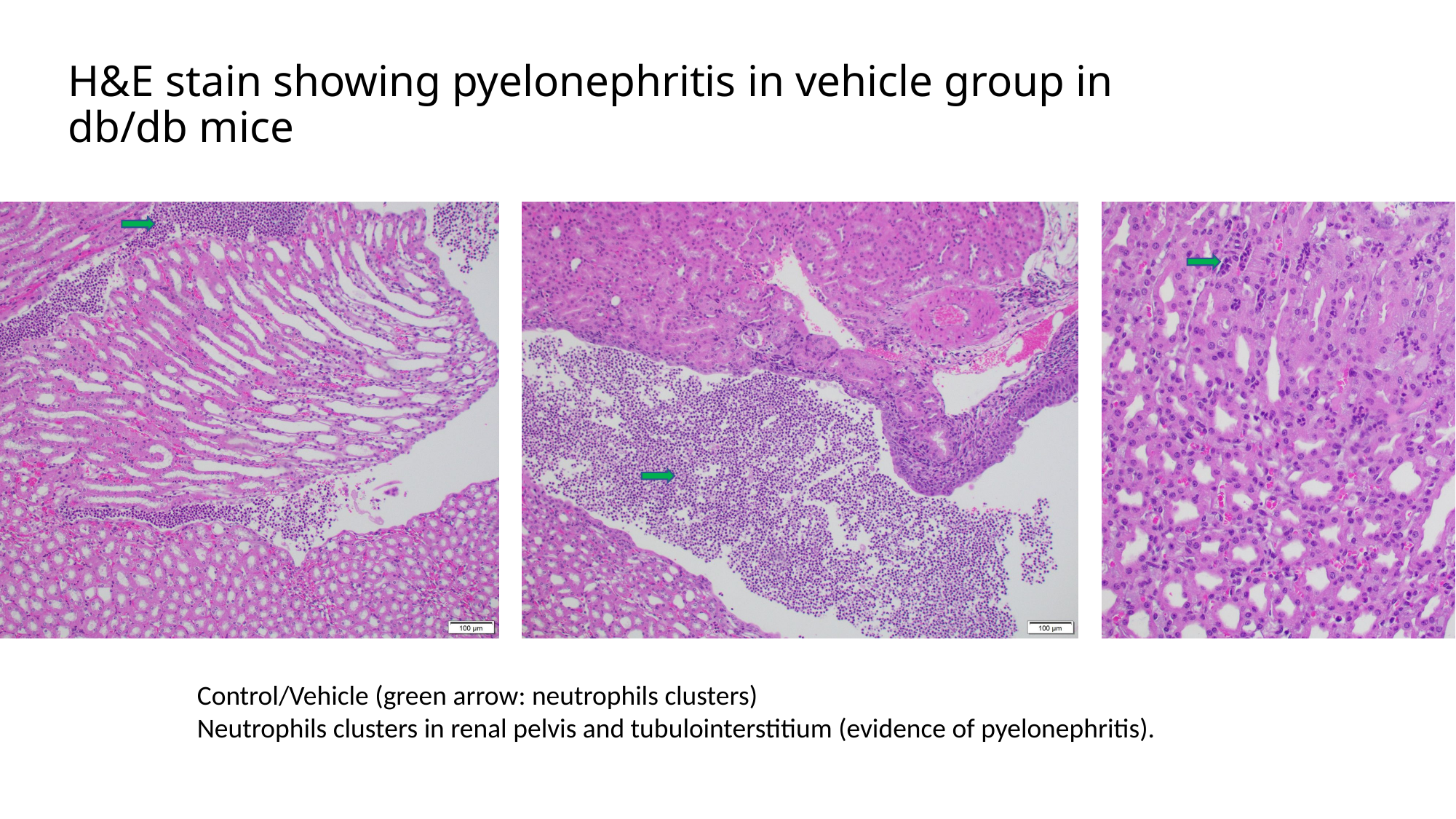

# H&E stain showing pyelonephritis in vehicle group in db/db mice
Control/Vehicle (green arrow: neutrophils clusters)
Neutrophils clusters in renal pelvis and tubulointerstitium (evidence of pyelonephritis).
